# Time‐resolved PET imaging using a joint dynamic reconstruction and motion estimation framework (DREME‐PET)

**DOI:** 10.1002/mp.70593

**Published:** 2026-08-04

**Authors:** Ruizhi Zuo, Hua‐Chieh Shao, Rameshwar Prasad, Jing Wang, You Zhang

**Affiliations:** ^1^ The Medical Artificial Intelligence and Automation (MAIA) Laboratory Department of Radiation Oncology University of Texas Southwestern Medical Center Dallas Texas USA

**Keywords:** image reconstruction, low‐count PET, motion model, real‐time motion tracking, time‐resolved imaging

## Abstract

**Background:**

Positron emission tomography (PET) plays a critical role in image‐guided radiotherapy by enabling accurate tumor localization and contouring. To account for respiration‐induced anatomical motion, time‐resolved PET imaging is highly desirable, as it captures dynamic anatomical variations to facilitate both disease diagnosis and treatment. However, reconstructing time‐resolved PET images is highly challenging due to the severely limited counts available within each short temporal frame (∼0.5 s) and the intrinsic ill‐posed nature of the inverse problem.

**Purpose:**

We proposed a time‐resolved PET imaging technique based on a dynamic reconstruction and motion estimation framework (DREME‐PET). The framework enables time‐resolved volumetric PET imaging and respiratory motion tracking directly from low‐count PET list‐mode data.

**Materials and methods:**

From a conventional PET scan, DREME‐PET reconstructs time‐resolved PET images while simultaneously generating a patient‐specific, data‐driven motion model. Based on the solved image and motion model by the time‐resolved reconstruction, DREME‐PET also enables real‐time PET and motion prediction based on subsequently acquired, minimal list‐mode data. Specifically, DREME‐PET employs spatial implicit neural representation (INR) to represent a reference volumetric PET image, and a corresponding low‐rank motion model to map the reference PET image to the time‐resolved PET sequence. The motion model comprises a trainable B‐spline‐based interpolant to represent the low‐rank motion basis components (MBC) and a CNN‐based motion encoder to derive the MBC scores from time‐resolved, low‐count list‐mode data. In addition to the count and motion variations observed in the training PET scan, the CNN encoder was trained with additional count‐ and motion‐augmentation to render it generalizable to a broader range of motion amplitudes and count variations (to account for the activity decay). DREME‐PET are evaluated with an XCAT simulation study, an in‐house physical phantom measurement, and a patient study.

**Results:**

DREME‐PET allows accurate time‐resolved PET reconstruction and motion modeling from a single PET scan, and enables real‐time image and motion predictions from subsequent list‐mode data acquisitions at a fast inference speed (28 ms per frame). For the XCAT simulation study, DREME‐PET achieves an average (±s.d.) image contrast relative error of 14.1% ± 3.0% and a tumor center‐of‐mass tracking error of 1.7 ± 0.8 mm. The physical phantom study shows an image contrast relative error of 7.4% ± 6.2% and a target center‐of‐mass tracking error of 0.5 ± 0.5 mm. The patient study shows a Pearson correlation higher than 0.91 and image contrast relative error of 3.6% ± 0.4%.

**Conclusions:**

DREME‐PET allows accurate time‐resolved PET reconstruction and motion modeling using a conventional, pre‐treatment‐delivery PET scan. Based on the derived motion model, it also enables real‐time, intra‐delivery PET imaging and motion tracking with minimal list‐mode data to benefit applications such as PET‐guided adaptive radiotherapy.

## 1 INTRODUCTION

Positron Emission Tomography (PET) is vital in clinical practice for diagnosis and detection of metabolic and functional abnormalities by qualifying the biochemical and physiological processes.^1^ Compared with computed tomography (CT), PET offers higher sensitivity for identifying and staging malignancies, enabling more accurate tumor delineation and improved treatment planning.^2^ However, respiratory motion in the thoracic and abdominal regions during PET acquisition leads to substantial organ and tumor displacement. Patient respiratory motion mainly has a cycle of 3–5 s,^3^ while the PET scan time per bed could reach 1–4 min. The spatial resolution of the current PET scanner is approximately 3–5 mm, while the motion of tumors in the lower lobes of the lung can be ∼30 mm in the cranial‐caudal direction.^4^ The motion introduces blurring and artifacts that degrade image quality, compromising lesion detectability, quantification accuracy, and downstream diagnostic and treatment decisions. Recently, RefleXion X1 (RefleXion Medical, Inc., Hayward, California, USA), a hybrid imaging‐therapy system that incorporates PET and CT into a linear accelerator (LINAC), has been developed to achieve biologically‐guided radiotherapy (BgRT). RefleXion rapidly reconstructs the PET images every 100 ms using the last 500 ms of information with a filtered back‐projection method. In this way, it can track the motion of the tumor and deliver the intended dose to the moving target. However, its simple reconstruction algorithm results in highly noisy images with limited tumor contrast, especially considering the limited counts from its small longitudinal field‐of‐view (5.2 cm).^5^ Therefore, for both diagnostic imaging and image‐guided radiotherapy applications, advanced imaging algorithms are needed to remove the motion artifacts, resolve the underlying motion, and generate high‐quality images.

To address the motion challenge, four‐dimensional PET (4D‐PET) was developed as a current solution.^6^ 4D‐PET sorts the sinogram/list‐mode data into different respiratory phases, enabling the reconstruction of a PET image series represented by semi‐static respiratory motion states. The resulting 4D‐PET images are less affected by respiratory motion but are not completely free of motion artifacts, due to phase sorting errors and intra‐phase residual motion.^7^ In addition, as each sorted 4D‐PET phase contains only a fraction of the total detected events, the resulting noisy images make accurate activity and motion quantification challenging.^8^ Post reconstruction registration^9^, ^10^ increases the signal‐to‐noise ratio (SNR) by using non‐rigid registration to align and average individual phases to a better‐quality, motion‐free image. However, these image‐domain registration‐based approaches treat image reconstruction and motion estimation as two separate steps, preventing full exploitation of the underlying PET raw data. In contrast, Kalantari et al.^11^ developed a raw‐data‐driven iterative algorithm for simultaneous motion estimation and image reconstruction (SMEIR) of 4D‐PET, using a deformation‐incorporated objective function for reconstruction. The corresponding results showed improved accuracy over image‐domain registration methods. However, 4D‐PET‐based approaches are intrinsically limited by phase sorting, which assumes a regular and periodic motion trajectory. Motion variations, including both inter‐cycle and intra‐cycle changes, cannot be resolved by 4D‐PET and are a major source of 4D sorting errors. To solve both regular and irregular motion, approaches were developed in the image and sinogram/list‐mode domains to capture motion from ultra‐fast acquisitions for *time‐resolved imaging*, which naturally resolves the motion without additional phase sorting. In the image domain, Bundschuh et al.^12^ extracted the center‐of‐mass (COM) for a selected region of interest from each 0.5 s PET reconstruction as a motion indicator to resolve the tumor movement. In the list‐mode domain, He et al.^13^ showed that axial motion will affect the total count rate per timeframe due to the axially non‐uniform sensitivity of 3D‐PET, which can be directly used as a surrogate for motion estimation. Spangler‐Bickell et al. developed a data‐driven registration‐based motion compensation method that uses 0.3–0.4 million events per frame, equivalent to roughly 0.5–1 s of acquisition time in typical clinical scans.^14^, ^15^ Munk et al. divided the list mode into a series of 1.0 s frames, and a center‐of‐distribution (COD) is calculated using time‐of‐flight (TOF) information of every list‐mode event. The COD estimates were subsequently used to track the underlying motion.^16^ Zeng et al. utilized an external stereovision camera to generate a point cloud up to 30 Hz to help the rigid‐body registration for PET.^17^ Although these methods could estimate rough motion, the ultrashort‐frame PET images inherently suffer from reduced image quality because each frame contains an extremely limited fraction of the total detected events. Some approaches further incorporate the estimated motion for motion‐compensated reconstruction to generate a reference image of better quality.^14^, ^15^, ^16^, ^17^ However, the correspondingly resolved motion by these approaches is mostly limited to bulk or rigid motion, failing to capture the spatiotemporal dynamics of deformable motion, which in turn affects the accuracy of the reconstructed reference image. Therefore, time‐resolved, high‐quality PET imaging that can faithfully capture the regular and irregular deformable motion is highly desired. Such PET images need to be accurately reconstructed from low‐count acquisitions of each temporal instance for anatomy and tumor localization to guide precision‐critical treatments like BgRT.

To mitigate the ill‐posed nature of the low‐count PET reconstruction, specific penalties or regularization were added to data fidelity terms of reconstruction methods to improve the PET quality, including the kernel method,^18^ total variation,^19^ and dictionary learning.^20^ However, due to the low SNR of the PET signal, kernel and total variation methods tend to over‐smooth the image,^21^, ^22^ and dictionary learning can be challenging as the feature sparsity assumption may not be guaranteed.^22^ In the past few years, deep learning (DL)‐based approaches have become popular in low‐count PET reconstruction, offering enhanced reconstruction efficiency and accuracy.^23^, ^24^, ^25^, ^26^, ^27^, ^28^ These methods typically operate on input data in the image, sinogram, or list‐mode domains, and are commonly trained or evaluated using only 1%–10% of the full‐count (standard‐dose) PET acquisition.^26^, ^27^, ^28^, ^29^, ^30^ Many of these DL solutions are based on image‐domain supervised learning approaches using dilate convolutional neural networks^30^ and generative adversarial networks,^26^ which need paired low‐ and full‐count PET images for network training. However, for these image‐domain only models, because the PET forward model is not explicitly included during inference, they may create image details inconsistent with sinograms. To address this challenge, deep unrolled methods are widely investigated by combining the physical characteristics of the PET imaging model with the powerful representation ability of deep neural networks. These frameworks transform iterative optimization methods into a series of concatenated, trainable neural networks to mimic the iterative reconstruction process. Hu et al. demonstrated two different deep unrolled methods based on the sinogram^25^ and list‐mode data^24^ using the primal dual network. Xie et al. integrated the agent‐driven foundation model in the deep unrolled network to achieve low‐count PET reconstruction.^28^ However, these deep learning‐based methods still require high‐quality PET images as a reference for training, which are not always available in clinical settings, particularly in cases affected by motion.^31^ Moreover, the absence of large, diverse PET datasets restricts the model generalizability of such supervised learning approaches, making it challenging for these methods to perform robustly across different scanners, patient populations, and clinical conditions. Recently, studies have demonstrated that incorporating high‐resolution Magnetic Resonance Imaging (MRI)^23^, ^29^, ^32^ or CT^27^, ^33^ as anatomical priors can enhance PET image quality by improving structural details and facilitating noise reduction. While these multi‐modality approaches have shown promising results, they also introduce significant drawbacks that may limit their clinical utility. Specifically, MRI often requires longer acquisition time and incurs higher hardware/imaging costs, which can reduce clinical accessibility and patient comfort. Research has also shown that prior images can unduly influence PET reconstruction, with MRI‐derived priors sometimes being overly apparent in the reconstructed PET images.^28^ Moreover, a precise alignment between the multi‐modality images and the PET can be challenging to achieve, especially for motion‐impacted scenarios.^31^

Implicit neural representation (INR)^34^ learning has emerged as a novel machine learning technique to map spatial coordinates to corresponding physical quantities (for instance, tracer activity concentrations). INR uses implicit continuous functions parameterized by multi‐layer perceptrons (MLPs) to represent volumetric images. INR's inductive bias^35^ suppresses noise and stabilizes PET reconstruction from low‐count sinograms, producing more consistent, artifact‐reduced images. The INR framework also allows model training/image reconstruction from raw data in a self‐supervised, “one‐shot” fashion, rendering it instance‐specific and thus not requiring a population‐based large dataset to train.^36^ Based on INR, Fan et al. developed the Implicit Neural Representation for Dynamic PET (IMREPET)^37^ method to achieve frame‐by‐frame PET signal variation reconstructions without the need for regularizations. Long et al. developed a prior‐driven INR,^38^ which incorporates image prior into conventional INR to help the reconstruction. Although these methods show promising reconstruction quality, they do not account for respiratory motion, which limits their clinical applicability to anatomical sites such as the liver or lung. Recently, INR has been extended to incorporate motion modeling, enabling joint image reconstruction and motion estimation across different imaging modalities.^36^, ^39^, ^40^, ^41^, ^42^ In the realm of cone‐beam computed tomography (CBCT), Shao et al. proposed a prior‐model‐free, spatial and temporal implicit neural representation framework (PMF‐STINR),^40^ which learns an on‐the‐fly motion model during time‐resolved CBCT reconstruction, and its effectiveness has been validated using multi‐institutional patient scans. Based on PMF‐STINR, Shao et al. further developed a joint dynamic reconstruction and motion estimation framework (DREME)^41^ for real‐time CBCT imaging and motion tracking. DREME employs a convolutional neural network (CNN) as a motion encoder to extract features from CBCT projections acquired at arbitrary angles to infer projection‐specific motion, effectively unifying the time‐resolved image reconstruction and real‐time motion estimation tasks. Specifically, it uses a pre‐treatment‐delivery setup CBCT scan to reconstruct a time‐resolved dynamic CBCT sequence, plus solving a motion encoder to infer *real‐time* CBCT and motion using new, intra‐treatment cone‐beam projections. The pre‐delivery model training and intra‐delivery imaging/motion monitoring work synergistically for precise image‐guided radiotherapy, as the patient‐specific model learned from an immediate pre‐delivery scan captures the most up‐to‐date anatomy and motion pattern for the subsequent intra‐delivery motion monitoring and treatment adaptation, minimizing potential challenges of model robustness/generalizability. Under a similar philosophy, DREME‐MR^42^ has been proposed to reconstruct dynamic volumetric MRIs and estimate time‐resolved respiratory and cardiac motion using multi‐coil k‐space signals via an MLP‐based motion encoder.

Inspired by the DREME framework, this study adapts and extends it to PET imaging by developing a joint dynamic reconstruction and motion estimation framework for time‐resolved and real‐time PET (DREME‐PET). While following the general formulation of Shao et al.’s studies, our approach incorporates PET‐specific modeling and addresses key challenges in PET imaging, including low‐count statistics, physical effects such as attenuation, the natural decay characteristics of PET acquisition, and real‐time processing requirements. DREME‐PET integrates time‐resolved volumetric PET reconstruction into a real‐time imaging and motion prediction framework. DREME‐PET performs two learning tasks within a single training session: (1) reconstructing a sequence of time‐resolved volumetric PET images from a conventional PET scan to obtain an up‐to‐date PET activity map and a patient‐specific motion model; and (2) simultaneously training a CNN‐based motion encoder during the time‐resolved PET reconstruction to enable future real‐time motion tracking and imaging based on new, minimal sinogram/list‐mode signals. For PET‐guided radiotherapy via a platform like Reflexion,^43^ the pre‐delivery, time‐resolved PET can help with motion quantification and target alignment, while the intra‐delivery, real‐time PET can facilitate treatment adjustment on‐the‐fly. Following a “one‐shot” learning strategy, DREME‐PET relies solely on the list‐mode data from a conventional PET scan, eliminating the requirement for a large‐scale, population‐based training dataset. For its first learning task, DREME‐PET employs INR to reconstruct a reference activity map and uses a deformable registration approach to align this reference with motion‐affected PET data through a learned low‐rank motion model. For the second task, a count and motion augmentation strategy is designed to fine‐tune the patient‐specific model to a broader range of unseen motion scenarios and count variation, to achieve robust motion estimation during real‐time imaging. DREME‐PET was validated using a digital phantom‐based simulation study, a physical phantom study, and a patient study. Compared with conventional expectation maximization (EM)‐based and learning‐based PET reconstruction methods, DREME‐PET achieves superior motion tracking accuracy and image quality.

## 2 MATERIALS AND METHODS

Time‐resolved PET imaging provides valuable insights for adaptive radiotherapy by enabling the real‐time tracking of radiotracers. A sequence of time‐resolved PET images, reconstructed from temporal frames of list‐mode data, comprehensively captures the spatiotemporal evolution of radiotracer distribution, which is critical for tumor detection and treatment plan optimization. A frame of list‐mode data is defined as a series of coincidence events collected within a short temporal frame, such that the intra‐frame movement could be negligible. In this study, for the whole PET acquisition, we collected a list‐mode dataset lasting about 90–150 s (per bed position). From this data, we were able to partition a time‐resolved sequence of 180–300 frames, providing a 0.5‐s temporal resolution that is sufficient for capturing respiratory motion. For the XCAT simulation and the patient study, ^18^F‐FDG (∼110‐min half‐life) was used, resulting in ∼1.57% decay over the 150‐s acquisition. In the physical phantom study, Ga‐68 (∼68‐min half‐life) was used, corresponding to ∼1.52% decay over the 90‐s acquisition. Therefore, activity changes due to radioactive decay are minimal during this period, and respiratory motion remains the dominant source of temporal variation across frames.

### 2.1 Time‐resolved PET reconstruction and motion modeling

To tackle the severely ill‐posed spatiotemporal inverse problem inherent in time‐resolved PET reconstruction, we designed a framework to simultaneously perform the image reconstruction and motion estimation using only the pre‐treatment‐delivery list‐mode data of a conventional PET scan as input. Because of the high intra‐frame spatial and temporal coherence of a series of PET data, we express the patient activity map I(x,t) of one timeframe (t) as a deformation of a reference image $I_{ref}\left(x\right)$. Specifically, the deformation is represented as a time‐dependent, list‐mode‐driven deformable vector field (DVF), that is,

(1) $$I\;\left(x,t\right)=I_{ref}\;\left(x+d\left(x,t\right)\right),$$

where $x\in R^{3}$ denote the voxel coordinates of the reconstruction. In the DREME‐PET, the $I_{ref}\left(x\right)$ is derived from a spatial INR to map the spatial coordinates x to radiotracer activity $I_{ref}\left(x\right)$. While some studies also use INR to represent the DVF,^44^ directly estimating voxel‐wise DVF for each frame could be highly ill‐posed and memory‐intensive. Alternatively, because respiratory motion is characterized by high spatial continuity and smooth temporal behavior, d(x,t) can be efficiently reduced in dimensionality and decomposed into separate spatial and temporal terms:

(2) $$d\;\left(x,t\right)=\sum_{i=1}^{L}w_{i}\left(t\right)\times e_{i}\left(x\right)\;,$$

where the spatial components $e_{i}\left(x\right)$ are the spatial basis functions (Motion Basis Components, MBC), modeled using a learnable B‐spline interpolant. And $w_{i}\left(t\right)$ denotes the temporal motion coefficients (MBC scores) that capture the motion state at each time frame, which are derived from a CNN‐based encoder. L denotes the number of spatial basis components in each Cartesian direction and is set as 3 in DREME‐PET (i.e., $e_{i,k}\left(x\right)$ and $w_{i,k}\left(x\right)$ where i=1,2,3 and k=x,y,z directions). It has been demonstrated that three levels (L=3) adequately model the dynamic respiratory motion in each direction due to its high spatiotemporal correlation.^41^, ^45^ This low‐rank representation substantially reduces memory usage and computational cost, which is particularly important for low‐count PET, where voxel‐wise motion estimation would otherwise be infeasible due to high noise and limited photon statistics. At the same time, it preserves essential motion dynamics while suppressing high‐frequency noise, thereby enhancing both reconstruction stability and temporal fidelity in time‐resolved PET images. An example of the resolved DVF from DREME‐PET is provided in the Supplementary Materials to further illustrate the represented motion by the learned motion basis components.

### 2.2 The DREME‐PET framework and dual‐task learning

**FIGURE 1:**
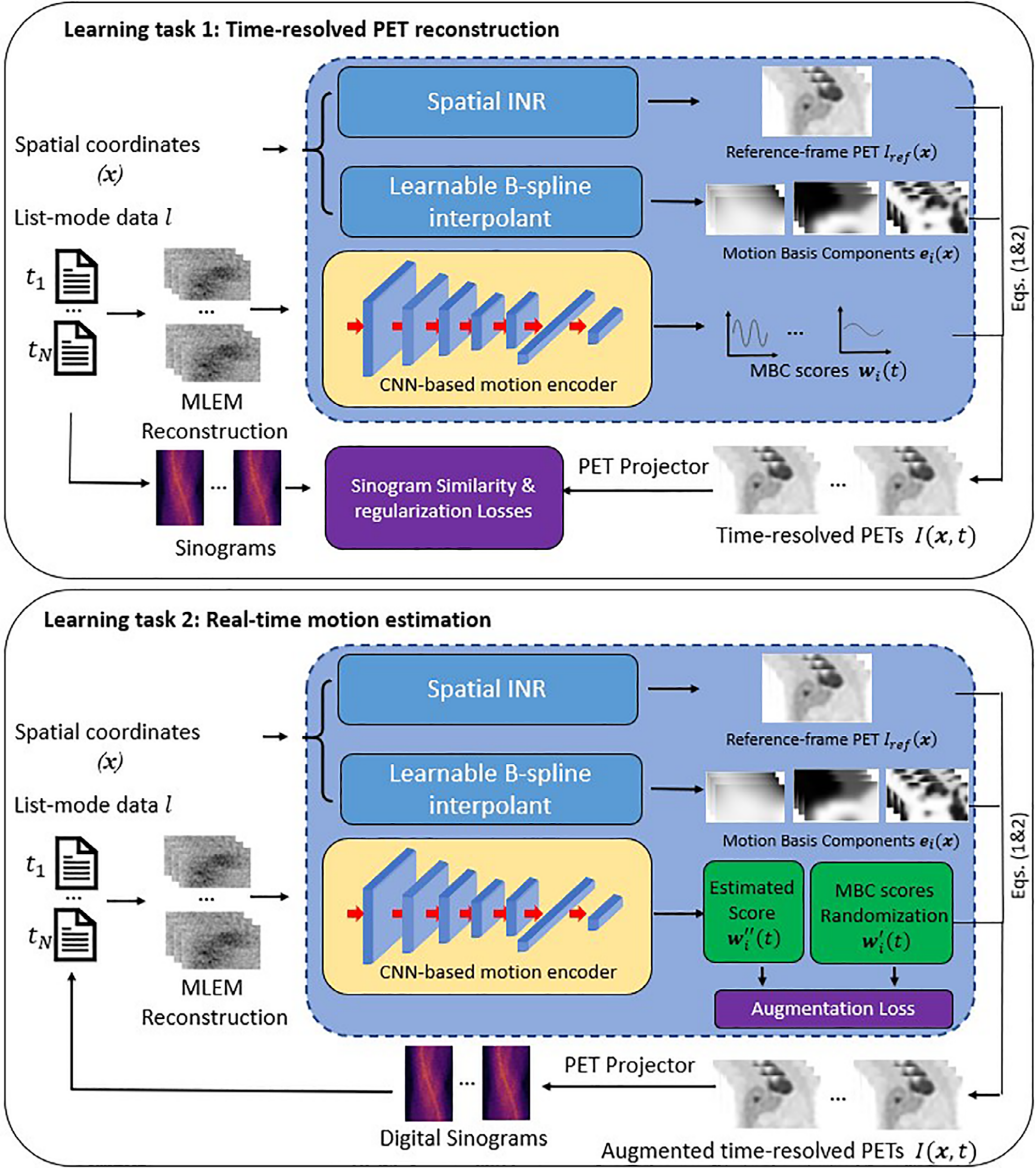
Overview of the DREME‐PET framework and dual‐task learning strategy. In the learning task 1 (upper panel), DREME‐PET reconstructs the time‐resolved PET images. Specifically, the spatial INR, learnable B‐spline interpolant, and CNN‐based motion encoder represent the reference image $I_{ref}\left(x\right)$, MBCs $e_{i}\left(x\right)$, and temporal motion coefficients $w_{i}\left(t\right)$, respectively. The motion encoder estimates the time‐varying motion coefficients $w_{i}\left(t\right)$ from the MLEM‐based PET volume reconstructed from the time‐resolved list‐mode data. This learning task is driven by the sinogram similarity loss and regularization losses. In the learning task 2 (lower panel), deformable motion augmentation is performed with randomized $w_{i}^{\prime }\left(t\right)$ to sample various motion. The motion‐augmented time‐resolved PET images are calculated to generate the digital sinograms and corresponding list‐mode data. In addition to motion augmentation, we also perform count augmentation by sub‐sampling the list‐mode data with a ratio of [0.5, 1], to make the CNN motion robust to activity decay. Based on the motion‐ and count‐augmented list‐mode data, the estimated scores 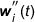 are compared with $w_{i}^{\prime }\left(t\right)$ to calculate the augmentation loss and fine‐tune the motion encoder. After training, the model could be applied to subsequent list‐mode measurements for real‐time motion estimation and PET imaging.

Figure 1 illustrates the workflow and network architecture of the DREME‐PET framework. DREME‐PET consists of a spatial INR, a learnable B‐spline interpolant, and a CNN‐based motion encoder. In the learning task 1: time‐resolved PET reconstruction (Figure 1, upper panel), the spatial INR and learnable B‐spline interpolant use spatial coordinates as input to generate $I_{ref}\left(x\right)$ and $e_{i}\left(x\right)$, respectively. In parallel, the list‐mode data of each 0.5 s is reconstructed to a 3D volume using a quick Maximum Likelihood Expectation Maximization (MLEM)^46^ module and then fed into the CNN‐based motion encoder to generate the MBC scores $w_{i}\left(t\right)$. Then, time‐resolved PETs are derived using Equations (1) and (2). During the training stage, the list‐mode data is first converted into 3D sinograms and compared with those projected from the time‐resolved PET volumes to compute a sinogram similarity loss. In addition, regularization losses are also applied to the $I_{ref}\left(x\right)$ and MBC $e_{i}\left(x\right)$, to improve the image/motion model quality and reduce the solution space. In the learning task 2: real‐time motion estimation (Figure 1, lower panel), we further train the motion encoder with deformable motion augmentation using randomized $w_{i}^{\prime }\left(t\right)$ to sample various motion. The augmented time‐resolved PET images are calculated, from which the (digital) sinograms and the corresponding list‐mode data are generated. In addition to motion augmentation, we also perform count augmentation by sub‐sampling the list‐mode data with a ratio of [0.5, 1], to make the CNN motion robust to activity decay. The estimated score 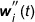, produced by the CNN motion encoder, is compared with $w_{i}^{\prime }\left(t\right)$ to compute the augmentation loss, which is used to fine‐tune the CNN motion encoder.

The spatial INR follows the concept of SIREN^47^ using MLPs with a periodic activation function. It consists of one input layer, three hidden layers, and one output layer. Both the input and hidden layers contain 32 feature channels, while the output layer has a single channel corresponding to the reconstructed voxel intensity. The learnable B‐spline interpolant produces the Motion Basis Components (MBCs, $e_{i}\left(x\right)$) by mapping voxel coordinates x through a differentiable B‐spline interpolation. The grid control points are learnable parameters with customized spatial levels for each Cartesian direction. DREME‐PET uses three levels and initializes a low‑level grid of size 6 × 6 × 6 and doubles the resolution at the second and third levels to refine motion details gradually. This hierarchical design enables the model to capture both global smooth motion and localized, potentially discontinuous motion patterns. In particular, higher‐resolution control point grids allow for more flexible local deformation, which helps approximate motion discontinuities or sharp transitions at organ boundaries.

To obtain the MBC scores ($w_{i}\left(t\right)$) from the CNN‐based motion encoder, an MLEM reconstruction is first performed on the list‐mode data of a temporal instance (0.5 s), before being passed to the encoder. The reconstruction is necessary, as the list‐mode data within a fixed time frame has a variable number of events, rendering the resulting uncertain data size unsuitable for direct input to the CNN. Alternatively, using the sinogram data computed from the list‐mode data as direct input to a CNN also offers a fixed data size.^48^ However, in low‐count PET, these projections are extremely sparse, resulting in a low signal‐to‐noise ratio. Consequently, the CNN encoder learns features that are easily dominated by noise and artifacts, which severely reduces its generalizability when applied to different imaging conditions, such as different counts of events and motion. The DREME‐PET method uses MLEM reconstruction on the list‐mode data primarily because of its stability as a CNN input. In addition, the reconstructed volume is a direct, dense representation of the activity distribution, making it easier for the CNN to track motion features. For low‐count list‐mode data, MLEM is computationally fast. In our implementation, MLEM only requires one iteration, which eliminates the need for the acceleration typically provided by Ordered Subset Expectation Maximization (OSEM).^49^ Because this reconstruction is primarily used for motion feature extraction rather than quantitative activity estimation, the MLEM step was implemented without scatter, random, or attenuation correction and without incorporating time‐of‐flight (TOF) information to enable faster computation. The reconstruction volume consists of 128 × 128 × 128 voxels, with an isotropic voxel size of 3 mm in all three spatial directions for the XCAT simulation and the patient study. For the physical phantom study, a voxel size of 1 mm in all three directions was used.″ The CNN motion encoder consists of five 3D convolution layers with 3 × 3 × 3 convolutional kernels. The feature map numbers for the layers are 32, 64, 128, 64, and 32, respectively. Following each convolution, the output is processed by a batch normalization layer and a Rectified Linear Unit (ReLU) activation function. After the final ReLU, the output is flattened and passed through a linear layer with nine output channels, which correspond to the nine MBC scores $w_{i,k}\left(t\right)$, where i=1,2,3 and k=x,y,z.

### 2.3 The loss function design and training strategy of DREME‐PET

#### 2.3.1 Learning task 1: Time‐resolved PET reconstruction

DREME‐PET uses a progressive training strategy with increasing training complexity and reconstruction resolution to improve learning efficiency and avoid overfitting. It consists of a low‐resolution stage (stage I) and a high‐resolution stage (stage II) with multiple substages.

During the first training substage (I‐a), the spatial INR model is initialized using an MLEM reconstruction from the list‐mode data of one time frame (0.5 s) to form an approximate PET $I_{app}\left(x\right)$. The image domain similarity loss is used in this stage by calculating the $L_{2}$ loss between $I_{ref}\left(x\right)$, which is voxelization from the spatial INR, and the $I_{app}\left(x\right)$,

(3) $$\;L_{sim}^{im}=\frac{1}{N_{voxel}}\;\sum_{N_{voxel}}{\left|I_{ref}\left(x\right)-I_{app}\left(x\right)\right|}^{2}$$

where $N_{voxel}$ denotes the total number of voxels in the PET image. This substage is mainly used to warm‐up the spatial INR and accelerate the convergence. In the next substage I‐b, the spatial INR and the motion model are simultaneously optimized with the sinogram similarity loss, $L_{sim}^{sino}$, between the sinogram p(l(t)) converted from the list‐mode data l(t), and the sinogram generated from the time‐resolved PET I(x,t) by the forward projector model P,

(4) $$\;L_{sim}^{sino}=\frac{1}{N_{batch}}\;\frac{1}{N_{pixel}^{S}}\sum_{N_{batch}}\sum_{N_{pixel}^{S}}{\left|p\left(l\left(t\right)\right)-P\left(I(x,t\right))\right|}^{2}$$

where $N_{batch}$ and $N_{pixel}^{S}$ are the batch size of the sinogram samples, and the pixel number of the sinogram, respectively. To build an accurate, physics‐based modeling for sinogram similarity loss, the forward projector model P is defined as follows:

(5) $$P\left(I\left(x,t\right)\right)=\mathrm{AFRI}\left(x,t\right)+s$$

where A is the attenuation correction matrix, F denotes the forward projection matrix, R represents the resolution model implemented as a Gaussian convolution operator, and s is an additive contamination term accounting for scatter and random events, and it is treated as fixed across all frames.^25^ Because the total acquisition duration in our study is relatively short, the underlying activity distribution changes minimally within this time window. Consequently, the scatter and random contributions are expected to vary only slightly across frames, making a fixed term a reasonable approximation for the proposed framework.

To ensure the training stability of the reference image, a total variation regularization is enforced on it to suppress the high‐frequency image noise while preserving the edges:

(6) $$\;L_{TV}=\frac{1}{N_{voxel}}\;\sum_{N_{voxel}}\left|\nabla I_{ref}\left(x\right)\right|,$$

where ∇ denotes the gradient operator. For the MBCs ($e_{i}\left(x\right)$), an ortho‐normality regularization loss is applied as

(7) $$\;L_{MBC}=\frac{1}{9}\;\sum_{k=x,y,z}\sum_{i=1}^{3}\left({\left|∥e_{i,k}{∥}^{2}-1\right|}^{2}+\sum_{j=i+1}^{3}{\left|e_{i,k}\cdot \;e_{j,k}\right|}^{2}\right),$$

where the subscript i=1,2,3 and k=x,y,z denotes the MBC level index, and the Cartesian components index, respectively.

In the high‐resolution training stage II, the reference activity map $I_{ref}\left(x\right)$ is first upsampled by a factor of 2 to accommodate the high‐resolution training. In the training substage II‐a, a 2x denser sampling of spatial coordinates is fed into spatial INR to ensure a smooth transition from low‐ to high‐resolution, and only the image‐domain $L_{sim}^{im}$ is applied as the loss. In the substage II‐b, the motion model is incorporated into training and $L_{sim}^{sino}$ is used as the similarity loss to further fine‐tune the spatial INR, while the B‐spline interpolant and the CNN motion encoder are frozen to stabilize the training. Subsequently, in the substage II‐c, both the B‐spline interpolant and the CNN motion encoder are unfrozen to fine‐tune the entire motion model to the high‐resolution scale.

#### 2.3.2 Learning task 2: Real‐time motion estimation for real‐time imaging

Additionally, another motion augmentation substage II‐d is introduced to avoid overfitting and expose the model to unseen motion scenarios. During this stage, we froze the spatial INR and the B‐spline interpolant to focus on improving the generalizability of the CNN motion encoder. We first generated augmented MBC scores $w_{i,k}^{\prime }\left(t\right)$ by random sampling using the following equation:

(8) $$w_{i,k}^{\prime }\;\left(t\right)=r_{1}\;\left(t\right)\times r_{2}\left(i,k\right)\times w_{i,k}\left(t\right),$$

where $r_{1}\in \left[0.5,\;2.0\right]$ and $r_{2}\in \left[0.5,\;1.6\right]$ are uniformly distributed random variables. These two random variables introduce variability into the temporal and spatial domains using different levels i and Cartesian directions k. The augmented MBC scores $w_{i,k}^{\prime }\left(t\right)$ can derive the randomized DVF through Equation (2) and then the corresponding deformation‐augmented PET image using Equation (1). From the deformation‐augmented PET image, we can simulate the sinogram and the corresponding list‐mode data and feed them into the MLEM module and the CNN‐motion encoder to infer the corresponding MBC scores 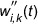. Instead of using all list‐mode data of each time instance (0.5 s), we used partial data to simulate natural PET radiotracer signal decay, enabling the model to generalize across varying numbers of events. In each training epoch, a random portion of the full list‐mode data at each time instance, ranging from 50% to 100%, was used for training. The augmentation loss is then defined as the mean squared error between the known scores $w_{i,k}^{\prime }\left(t\right)$ and CNN‐predicted scores 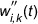:

(9)

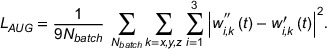




Additionally, in the list‐mode‐based method,^13^ the number of events could be used as a feature to distinguish the motion states due to the axial non‐uniform sensitivity of the PET scanner. By randomly varying the number of events used in each iteration, the CNN motion encoder is forced to learn motion patterns from the spatial structure of activity distribution rather than from the event counts, thereby preventing the model from relying on this unintended correlation.

In summary, in each stage of the DREME‐PET, the total loss function is a weighted sum of the above loss functions. The training strategy is summarized in Table 1 with more details such as the learning rate (LR) and the epoch number provided. For training stage I‐b, II‐b, and II‐c, the weighting factors of $L_{sim}^{sino},L_{TV},$ and $L_{MBC}$ are 1, 2 × 10^−7^, and 1, respectively. N/A means that the learning rate is not applicable for the component, as the affected network component is frozen in the corresponding stage. The choice of spatial resolution is mainly restricted by the GPU memory and can be improved when more computational resource becomes available.

**TABLE 1:** The multi‐resolution training strategy of DREME‐PET with different stages and loss functions.

Training stage	I‐a	I‐b	II‐a	II‐b	II‐c	II‐d
Number of epochs	4000	4000	1000	1000	1000	1000
Spatial resolution (mm^3^)	6 × 6 × 6			3 × 3 × 3		
LR of spatial INR	2 × 10^−4^	2 × 10^−5^	1 × 10^−6^	1 × 10^−6^	1 × 10^−6^	N/A
LR of B‐spline interpolant	N/A	2 × 10^−5^	N/A	N/A	1 × 10^−5^	N/A
LR of the motion encoder	N/A	2 × 10^−5^	N/A	N/A	1 × 10^−5^	1 × 10^−5^
Loss function(s) used	$L_{sim}^{im}$	$L_{sim}^{sino},L_{TV},L_{MBC}$	$L_{sim}^{im}$	$L_{sim}^{sino},L_{TV}$	$L_{sim}^{sino},L_{TV},L_{MBC}$	$L_{AUG}$

### 2.4 On‐board real‐time imaging and motion tracking

After dual‐task learning, DREME‐PET yields a reference activity map $I_{ref}\left(x\right)$ and the corresponding motion model, which can be directly applied during the subsequent treatment delivery to achieve real‐time volumetric imaging and motion tracking. When deployed during radiation treatment, DREME‐PET will continually acquire online PET list‐mode data. The data will then be used to infer $w_{i}\left(t\right)$ and generate real‐time DVFs from the pre‐computed $I_{ref}\left(x\right)$ and $e_{i}\left(x\right)$, following Equation (2). Finally, the DVF is applied to the reference activity map to derive the real‐time PET volume. If the reference activity map is replaced with a target contour, the DREME‐PET can be used to achieve markerless target localization. Note that the conversion between list‐mode and 3D sinogram is only required during the training stage and is not necessary during the on‐board inference.

### 2.5 Evaluation datasets and schemes

We evaluated DREME‐PET using the extended cardiac torso (XCAT) digital phantom,^50^ a physical phantom, and a patient scan. The XCAT phantom provides ground‐truth images for each motion frame, enabling quantitative assessment of image reconstruction and motion estimation accuracy. The physical phantom study employed two point sources mounted on a user‐programmed motion translation stage, allowing validation under controlled, realistic motion scenarios that mimic patient respiratory motion.

#### 2.5.1 XCAT simulation study

To evaluate the ability of DREME‐PET to capture different types of motion variations in a “one‐shot” learning manner, we simulated seven XCAT motion scenarios (C1‐C7) featuring irregular variations in amplitude, frequency, and baseline shift. In addition to these designed variations, the XCAT phantom inherently models different organs and anatomical structures with distinct motion components, resulting in complex motion patterns such as relative motion at organ interfaces (e.g., sliding of the lungs against the ribs).^50^ All scenarios include the motion in both superior‐anterior (SI) and anterior‐posterior (AP) directions. In order to evaluate the motion tracking accuracy, a spherical tumor was inserted in the liver with a diameter of 30 mm and served as the tracking target. The XCAT volumes contain 128 × 128 × 128 voxels with a 3 × 3 × 3 mm^3^ resolution, covering the abdominal region. The tracer activity distribution of XCAT simulation was generated based on the typical uptake patterns of ^18^F‐FDG.^51^ Specifically, we assigned representative relative activity values to several organs and tissues (arbitrary unit): body background = 2, liver = 5, tumor = 15, myocardium = 12, and kidney = 7. These values were selected to simulate the realistic FDG uptake contrasts among different anatomical regions. An attenuation map is also generated from XCAT for attenuation correction, with a well‐defined registration between the attenuation map and the PET image. In each scenario, the motion lasted 150 s, and we generated a sequence of 300 volumetric frames, yielding a temporal resolution of 0.5 s to capture the regular/irregular respiratory motion. Sinogram and corresponding list‐mode data sequences were simulated from each frame through forward projection with additional Poisson noise incorporated. The projectors were provided by Parallelproj^52^ which supports customizable PET scanner geometries and GPU‐based forward and backward projection implementations, and we followed its simulated PET scanner configuration. Each 0.5 s frame contained approximately 0.2 million total events, with ∼30% of the counts contributed by random noise and contamination. To evaluate the accuracy of DREME‐PET, we trained DREME‐PET using the raw data from the C2 scenario, and used the C1‐C7 raw data for time‐resolved reconstruction (for C2) and real‐time imaging/motion prediction assessment (for C1, and C3‐C7).

The performance of DREME‐PET was evaluated using several quantitative metrics. To assess the radiotracer reconstruction accuracy, we calculated the standard uptake value (SUV) and image contrast. SUV is widely used because it reduces the variability associated with differences in patient size and injected tracer dose^53^ and is computed as

(10) $$SUV=\frac{activity\;concentration\;in\;an\;ROI\;\left(MBq\;ml^{-1}\right)}{injected\;dose\left(MBq\right)/\;body\;weight\left(g\right)}.$$

SUVs were determined assuming a 74 kg patient and 370 MBq of injected FDG, routinely used in clinical practice, with the tumor set as the region of interest (ROI). Image contrast was calculated as the ratio of the activity concentration in the tumor to that in a background region selected from the healthy liver. We calculated the relative error (RE) between the DREME‐PET's SUV/image contrast and those of the “ground‐truth” (GT) XCATs:

(11) $$RE_{\mathrm{SUV}}=\frac{1}{N_{t}}\sum_{i=1}^{N_{t}}\left|\frac{\mathrm{SU}V_{\mathrm{DREME}}^{i}-\mathrm{SU}V_{\mathrm{GT}}^{i}}{\mathrm{SU}V_{\mathrm{GT}}^{i}}\right|\times 100\%,$$

(12) $$RE_{\mathrm{cont}\mathrm{rast}}=\frac{1}{N_{t}}\sum_{i=1}^{N_{t}}\left|\frac{\mathrm{Cont}\mathrm{ras}t_{\mathrm{DREME}}^{i}-\mathrm{Cont}\mathrm{ras}t_{\mathrm{GT}}^{i}}{\mathrm{Cont}\mathrm{ras}t_{\mathrm{GT}}^{i}}\right|\times 100\%,$$

in which $N_{t}$ is the total number of frames used for evaluation. To assess the motion tracking performance, the “ground‐truth” tumor segmentations were derived from the “ground‐truth” XCAT images through auto‐thresholding. For DREME‐PET, we first segmented the tumor of $I_{ref}$ using a threshold‐based method and then applied frame‐specific DVFs (d(x,t)) to propagate the reference tumor segmentation to each frame. For each frame, the resulting center of mass of DREME‐PET's tumor segmentation was compared with the XCAT “ground truth” to derive the center‐of‐mass error (COME).^54^ In addition, the Dice similarity coefficient (DSC)^55^ was calculated based on the tumor segmentation to further quantify the volume tracking accuracy.

#### 2.5.2 Physical phantom study

An in‐house physical phantom with two point sources was used for testing time‐resolved PET imaging. Two 3.7MBq Ga‐68 point sources (Eckert & Ziegler, Braunschweig, Germany) were mounted on a CIRS motion stage (Sun Nuclear, Melbourne, Florida, USA) to perform user‐programmed one‐dimensional longitudinal motion. Four motion scenarios (S1–S4) were implemented to mimic patient respiratory motion with variations in motion range, frequency, and baseline shift. The details of these scenarios are summarized in Table 2.

**TABLE 2:** Parameters and characteristics of motion scenarios (S1‐S4) in the physical phantom study.

Motion scenario	Motion range (mm)	Cycle period (s)	Motion characteristics
S1	20	3.6	Regular sinusoidal wave
S2	20	5	Regular sinusoidal wave
S3	30	5	Baseline shift
S4	30	5	Baseline shift and amplitude variation

For each scenario, list‐mode data were acquired over 90 s using a GE Omni Legend 32 PET/CT system (GE Healthcare, Chicago, Illinois, USA).^56^ The raw dataset includes time markers, allowing the data to be divided into 180 sequential time frames, each containing the events accumulated over 0.5 s. Owing to the high sensitivity of the scanner, each time frame contains approximately 83,000 events, which is sufficient to reconstruct the two point sources. To mimic low‐count conditions, we randomly selected 3% of the total events in each frame for reconstruction and evaluation. During training of the proposed network, the list‐mode data are converted into sinograms to compute the sinogram similarity loss. In addition, to account for the GPU memory restriction, we limited the oblique planes to a maximum ring difference of ≤16, striking a balance between GPU memory usage and reconstruction resolution. After this processing, each time frame contains approximately 550 events for reconstruction. During reconstruction, the volumes were defined with 128 × 128 × 128 voxels at a 1 × 1 × 1 mm^3^ resolution to capture the point sources’ motion. Correspondingly, in DREME‐PET, the spatial resolution is set as 2 × 2 × 2 mm^3^ and 1 × 1 × 1 mm^3^ in low‐ and high‐resolution training stages, respectively. We did not perform the attenuation correction in this physical phantom study due to its simple geometry.

To evaluate the motion tracking and reconstruction accuracy of DREME‐PET in this phantom study, we utilized COME, Pearson correlation coefficient^57^ and $RE_{contrast}$ as metrics. For COME, we first segmented the two point sources in each frame and calculated the center of mass for each. A motion curve over time was then extracted from these center‐of‐mass coordinates, which was compared with the input “ground‐truth” motion curve to compute the COME. The Pearson correlation coefficients were calculated to measure the similarity of the derived curve and the input curve. For $RE_{contrast}$, the reconstruction contrast is defined as the ratio of activity between two points sources. As the two sources were produced at different times, they have decayed to different activity levels. In addition to S1–S4, we also acquired a reference scenario in which the point sources remained stationary over 90 s. A vendor‐provided algorithm, using all events collected for 90 s, was used for reconstruction, and the contrast between the two sources served as the “ground truth”.

#### 2.5.3 Patient study

A patient study was conducted to evaluate the robustness of time‐resolved PET imaging in a real clinical setting. The patient was injected with FDG, and a 150‐s list‐mode dataset was acquired 1 h post‐injection using the same PET/CT system as in the phantom study. The first 75 s of data were divided into 150 frames (0.5 s per frame) and used for time‐resolved reconstruction, while the remaining 75 s were used to assess real‐time prediction performance. Owing to the high sensitivity of the scanner, each timeframe contains around 1.4 million events. To address GPU memory constraints, oblique planes were limited to a maximum ring difference of ≤10, resulting in approximately 0.3 million events per timeframe. The volumes were reconstructed as 128 × 128 × 128 voxels at a 3 × 3 × 3 mm^3^ resolution to capture liver motion. An attenuation correction CT image acquired from the same system is used to implement the attenuation correction. The CT image was rigidly registered to the PET reference image.

To quantitively evaluate the motion tracking and reconstruction accuracy of DREME‐PET in this patient study, we use the Pearson correlation coefficient, and $RE_{contrast}$ as metrics. We first segmented the liver from the reference PET image and propagated the liver mask to all timeframes using the resolved DVFs. This allowed us to extract the specific liver motion state for each timeframe. Due to the lack of the “ground truth”, the number of events within each timeframe, after decay correction, was used as the surrogate of the respiratory motion, as in prior studies.^13^ The Pearson correlation coefficient was calculated to show the similarity between the extracted liver motion and the surrogate curve. For $RE_{contrast}$,, it is defined as the ratio of activity between the central region of the liver and the chest wall soft tissue. The “ground truth” is defined using vendor‐provided reconstruction, as the liver center is relatively stable under motion and the chest wall is minimally affected by motion.

#### 2.5.4 Comparison and parameter studies

We further compared the performance of DREME‐PET with several representative reconstruction methods to establish a comprehensive evaluation of its effectiveness. For the conventional MLEM‐based methods, we included single‐frame 3D‐PET and 4D‐PET reconstructions for comparison. EM‐based methods are statistically grounded and widely regarded as the standard reference for PET reconstruction due to their well‐established accuracy. These methods can also be efficiently implemented using parallel computing and are routinely adopted in clinical practice. Both methods were implemented with 2 MLEM iterations. The single‐frame 3D‐PET uses the list‐mode data within one frame to reconstruct the volumetric PET image. For the 4D‐PET, we first phase‐sorted the list‐mode data into 10 frames and reconstructed the PET images for each phase. During evaluation, the motion phase of 4D‐PET that most closely matched the “ground‐truth” motion state was selected for comparison. Each motion phase of 4D‐PET includes approximately 6–9 million events. Due to the limited number of events per phase, the reconstructed images are inherently blurred and noisy. To address this limitation, we implemented ano enhanced 4D‐PET (E4D‐PET) method for comparison. In E4D‐PET, motion estimation is incorporated directly into the forward/backward projection during reconstruction. This approach jointly estimates a reference PET image along with a deformation vector field (DVF) for each phase, enabling the use of all available counts in the measurements. Specifically, the raw list‐mode data were first sorted into ten phases, yielding ten corresponding sinograms. These data were then processed within the DREME‐PET framework, with the input to the CNN‐based motion encoder replaced by the corresponding phase index for each frame. We also included deep learning‐based methods for comparison. Noise2Void (N2V)^58^ learns to denoise images without clean targets by masking pixels and training a network to predict each masked pixel from its surrounding context, forcing the model to learn the underlying signal rather than the noise. We trained a Noise2Void model on the single‐frame 3D‐PET images to improve their SNR. Additionally, A direct low‐count to high‐count image mapping was also implemented using a U‐Net^59^ architecture with 3D operators as a supervised learning method (UNET) for comparison. As true high‐count images within short time frames are not available clinically, the 3D‐PET images served as the low‐count input, while the corresponding phase‐sorted 4D‐PET reconstructions provided the higher‐count targets. This pairing offers a practical surrogate that captures realistic differences in count statistics and motion‐induced variability. UNET and N2V offer computationally efficient post‐processing at deployment and represent a common deep learning strategy for image enhancement and denoising. Therefore, both EM‐based and DL‐based methods provide meaningful comparisons in both reconstruction accuracy and runtime performance.

To evaluate the robustness of the proposed network, we conducted two parameter studies using the XCAT simulation. In the first study, we varied the number of detected events per time frame and reconstructed time‐resolved PET images using 0.1 million, 0.2 million, 0.5 million, and 1 million events. The varying counts study allowed us to systematically assess how the quantitative metrics change as a function of the available count statistics. By analyzing performance across these different event levels, we aimed to determine the network's stability under varying noise conditions and its sensitivity to reduced photon statistics. We further performed a second parameter study to evaluate the network's robustness to activity decay during acquisition. In this experiment, the network was trained using 0.2 million events per time frame but tested with reduced event statistics corresponding to 100%, 90%, 80%, 70%, and 60% of the training level. This setup simulates the gradual decrease in detected counts caused by radioactive decay and enables assessment of how well the network generalizes when the test data contain substantially fewer events than the training data.

## 3 RESULTS

### 3.1 The XCAT simulation study

**FIGURE 2:**
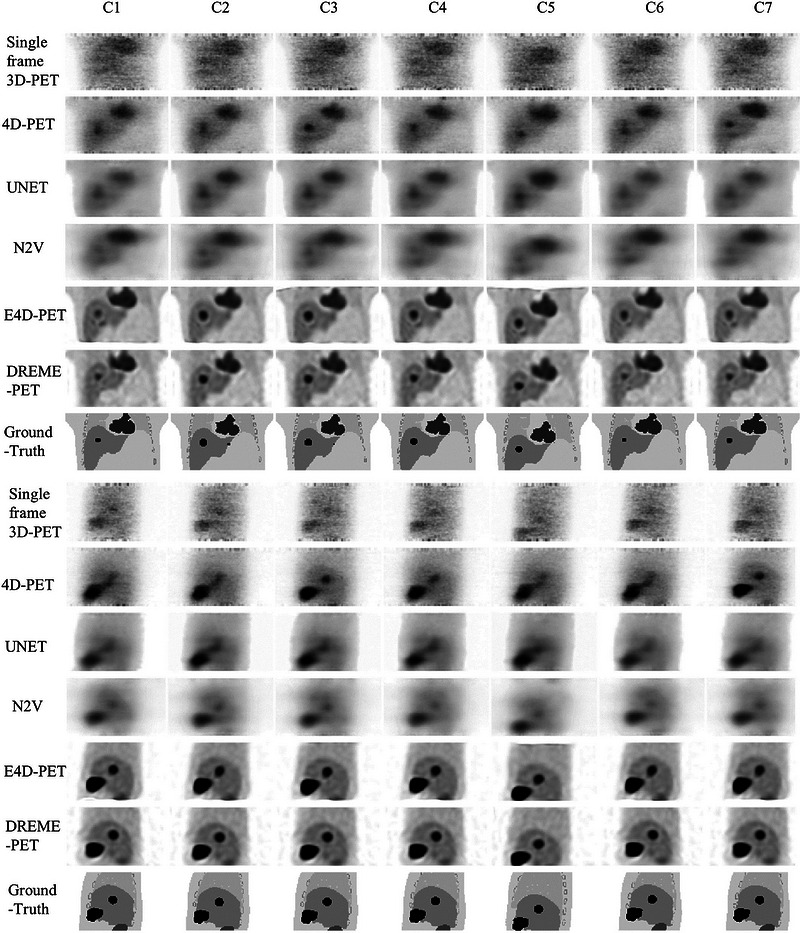
Comparison of reconstructed PET images at one frame from different scenarios (C1‐C7) for the XCAT study, between single‐frame 3D‐PET, 4D‐PET, UNET, Noise2Void(N2V), E4D‐PET, DREME‐PET, and “ground truth”. The first seven rows and last seven rows show the coronal and sagittal views, respectively. For 4D‐PET and E4D‐PET, the image with its phase matching that of the shown time frame is selected for display and comparison. DREME‐PET simultaneously learns the patient activity map and a motion model using all available raw data (of scenario C2 in this study), leading to the reconstructed PET images with minimal motion‐induced artifacts. As DREME‐PET was trained on C2, the other scenarios (C1, C3‐C7) show real‐time images predicted from signals of 0.5 s acquisitions, demonstrating DREME‐PET's real‐time imaging prediction capability.

Figure 2 presents a representative frame of time‐resolved PET images reconstructed by DREME‐PET and all comparison methods for the XCAT simulation study, shown in both coronal and sagittal views. The “ground truth” is also included as a reference. For 4D‐PET and E4D‐PET, the image with its phase matching that of the shown time frame is selected for display and comparison. As mentioned in Section 2.5.1, the DREME‐PET model was trained on scenario C2 and evaluated across all seven motion scenarios (C1–C7). *Thus, the DREME‐PET results of C2 indicate the accuracy of time‐resolved reconstruction, and those of the other scenarios indicate the accuracy of real‐time imaging/motion predictions*. The single‐frame 3D‐PET suffers from excessive noise and artifacts, as it is reconstructed from a limited number of events (0.2 million, containing ∼30% noise). Due to accumulated events from phase sorting and binning, the 4D‐PET provides more structural details, but the tumor shape appears distorted due to irregular motion (baseline shifts for C2). The results from the U‐Net and N2V methods, which benefit from learning‐based training, exhibit reduced noise compared to non‐learning approaches. However, distinguishing tissue boundaries remains challenging for their outputs. E4D‐PET benefits from utilizing all available events and therefore provides sharper structural details. However, because retrospective phase sorting assumes regular motion, its reconstruction can become erroneous in the presence of irregular motion. For example, the tumors in C6 and C7, which are expected to exhibit a circular morphology, appear distorted in the reconstruction by E4D‐PET. In contrast, DREME‐PET simultaneously trains the patient activity map and motion model, allowing it to use all collected events (of scenario C2 in this study) for reconstruction rather than only a fraction of the events, in contrast to other methods. This leads to minimal motion‐induced artifacts and sharper structure edges, as reflected in both the time‐resolved reconstruction (C2) and real‐time prediction (C1, C3–C7) results.

A quantitative evaluation of the XCAT simulation study across all reconstruction methods and seven scenarios is summarized in Tables 3 and 4. The single‐frame 3D‐PET is excluded due to its poor performance. Table 3 compares the relative error of SUV and contrast for the reconstructed PET images, where DREME‐PET outperforms all other methods on both metrics. By utilizing all acquired list‐mode data of a conventional PET scan during training, DREME‐PET achieves more accurate structural details and reduces motion blur. The resulting high‐quality reference image and motion model from time‐resolved reconstruction (for C2) subsequently benefited the real‐time motion/image predictions (for C1, C3–C7). Table 4 presents the COME and DSC for each scenario to assess the tumor‐tracking performance. DREME‐PET achieves sub‐voxel accuracy and best results among all methods. The other four methods perform worse, as the blurred tumor boundaries make accurate segmentation challenging from each frame. All Wilcoxon signed‐rank tests of four metrics between DREME‐PET and other methods yielded *p* values < 10^−3^.

**TABLE 3:** Relative error of SUV and contrast as accuracy measures of the reconstrued time‐resolved PET images for the XCAT simulation study using different methods.

XCAT scenarios	4D‐PET $RE_{SUV}$ ↓ $RE_{Contrast}$↓	UNET $RE_{SUV}$↓ $RE_{Contrast}$↓	Noise2Void $RE_{SUV}$↓ $RE_{Contrast}$↓	E4D‐PET $RE_{SUV}$ ↓ $RE_{Contrast}$↓	DREME‐PET $RE_{SUV}$↓ $RE_{Contrast}$↓
C1	48.5% ± 9.5%	42.7% ± 3.2%	56.7% ± 1.1%	21.9% ± 4.1%	**17.5% ± 2.2%**
	51.7% ± 9.4%	49.9% ± 3.2%	59.9% ± 1.5%	22.6% ± 4.9%	**13.2% ± 4.1%**
C2	46.7% ± 5.9%	43.4% ± 2.3%	57.1% ± 1.3%	26.0% ± 4.5%	**16.7% ± 1.6%**
	51.0% ± 5.4%	50.7% ± 2.1%	59.4% ± 1.4%	26.8% ± 5.9%	**15.2% ± 1.4%**
C3	43.2% ± 7.5%	43.3% ± 1.2%	56.8% ± 1.1%	21.5% ± 2.5%	**17.6% ± 1.3%**
	46.8% ± 7.2%	51.0% ± 1.3%	60.1% ± 1.2%	23.8% ± 2.9%	**15.0% ± 1.7%**
C4	45.5% ± 7.2%	43.2% ± 1.9%	56.7% ± 1.1%	21.9% ± 3.5%	**17.5% ± 1.5%**
	49.1% ± 7.6%	50.6% ± 2.0%	59.5% ± 1.4%	24.1% ± 5.3%	**15.0% ± 2.0%**
C5	48.8% ± 8.0%	42.7% ± 3.4%	56.8% ± 1.2%	20.9% ± 1.7%	**17.1% ± 1.9%**
	52.8% ± 7.6%	49.7% ± 3.4%	59.5% ± 1.3%	20.6% ± 4.4%	**13.1% ± 4.5%**
C6	48.8% ± 8.8%	42.7% ± 3.2%	56.7% ± 1.2%	26.1% ± 6.1%	**17.7% ± 1.9%**
	53.3% ± 8.3%	50.0% ± 3.2%	59.6% ± 1.3%	27.3% ± 6.5%	**13.1% ± 4.5%**
C7	46.3% ± 7.6%	43.0% ± 2.1%	56.9% ± 1.1%	26.0% ± 5.8%	**17.3% ± 2.0%**
	50.4% ± 7.8%	50.6% ± 2.3%	59.7% ± 1.3%	28.2% ± 5.9%	**14.3% ± 2.8%**

*Note*: For the C2 scenario, the results quantify the image accuracy of time‐resolved reconstruction, as the DREME‐PET model was trained using all the raw data of C2. For the other scenarios (C1, C3‐C7), the results quantify the image accuracy of real‐time predictions, as the images were directly inferred based on the model trained on C2. The arrows point to the direction of improved accuracy.

**TABLE 4:** Center‐of‐mass error (COME) and dice similarity coefficient (DSC) as accuracy metrics of the solved tumor motion for the XCAT simulation study.

XCAT scenarios	4D‐PET COME (mm) ↓ DSC ↑	UNET COME (mm) ↓ DSC ↑	Noise2Void COME (mm) ↓ DSC ↑	E4D‐PET COME (mm) ↓ DSC ↑	DREME‐PET COME (mm) ↓ DSC ↑
C1	6.8 ± 0.4	5.5 ± 2.1	8.3 ± 7.9	3.7 ± 2.8	**1.8 ± 0.9**
	0.410 ± 0.210	0.663 ± 0.084	0.409 ± 0.368	0.823 ± 0.111	**0.882 ± 0.041**
C2	6.6 ± 0.2	5.3 ± 1.8	7.8 ± 7.7	4.8 ± 3.4	**1.6 ± 0.8**
	0.458 ± 0.194	0.706 ± 0.08	0.424 ± 0.369	0.774 ± 0.129	**0.889 ± 0.034**
C3	7.1 ± 0.1	5.1 ± 1.0	9.2 ± 7.7	3.3 ± 2.1	**1.7 ± 0.7**
	0.533 ± 0.127	0.782 ± 0.050	0.349 ± 0.376	0.841 ± 0.085	**0.888 ± 0.030**
C4	7.0 ± 0.2	5.3 ± 1.6	7.9 ± 7.7	3.2 ± 2.5	**1.7 ± 0.7**
	0.474 ± 0.178	0.720 ± 0.067	0.418 ± 0.376	0.836 ± 0.053	**0.885 ± 0.037**
C5	6.8 ± 0.5	5.4 ± 2.2	8.2 ± 8.0	2.7 ± 1.9	**1.7 ± 0.9**
	0.385 ± 0.210	0.661 ± 0.099	0.409 ± 0.369	0.852 ± 0.066	**0.887 ± 0.034**
C6	6.7 ± 0.4	5.6 ± 2.1	9.0 ± 8.0	4.9 ± 4.3	**1.8 ± 1.0**
	0.389 ± 0.210	0.676 ± 0.077	0.371 ± 0.370	0.770 ± 0.159	**0.880 ± 0.042**
C7	7.1 ± 0.3	5.0 ± 1.7	8.3 ± 7.7	4.5 ± 4.2	**1.8 ± 0.9**
	0.471 ± 0.170	0.726 ± 0.080	0.404 ± 0.371	0.780 ± 0.151	**0.884 ± 0.038**

*Note*: For the C2 scenario, the results quantify the motion accuracy of time‐resolved reconstruction, as the DREME‐PET model was trained using all the raw data of C2. For the other scenarios (C1, C3–C7), the results quantify the motion accuracy of real‐time predictions, as the motion was directly inferred based on the model trained on C2. The arrows point to the direction of improved accuracy.

**FIGURE 3:**
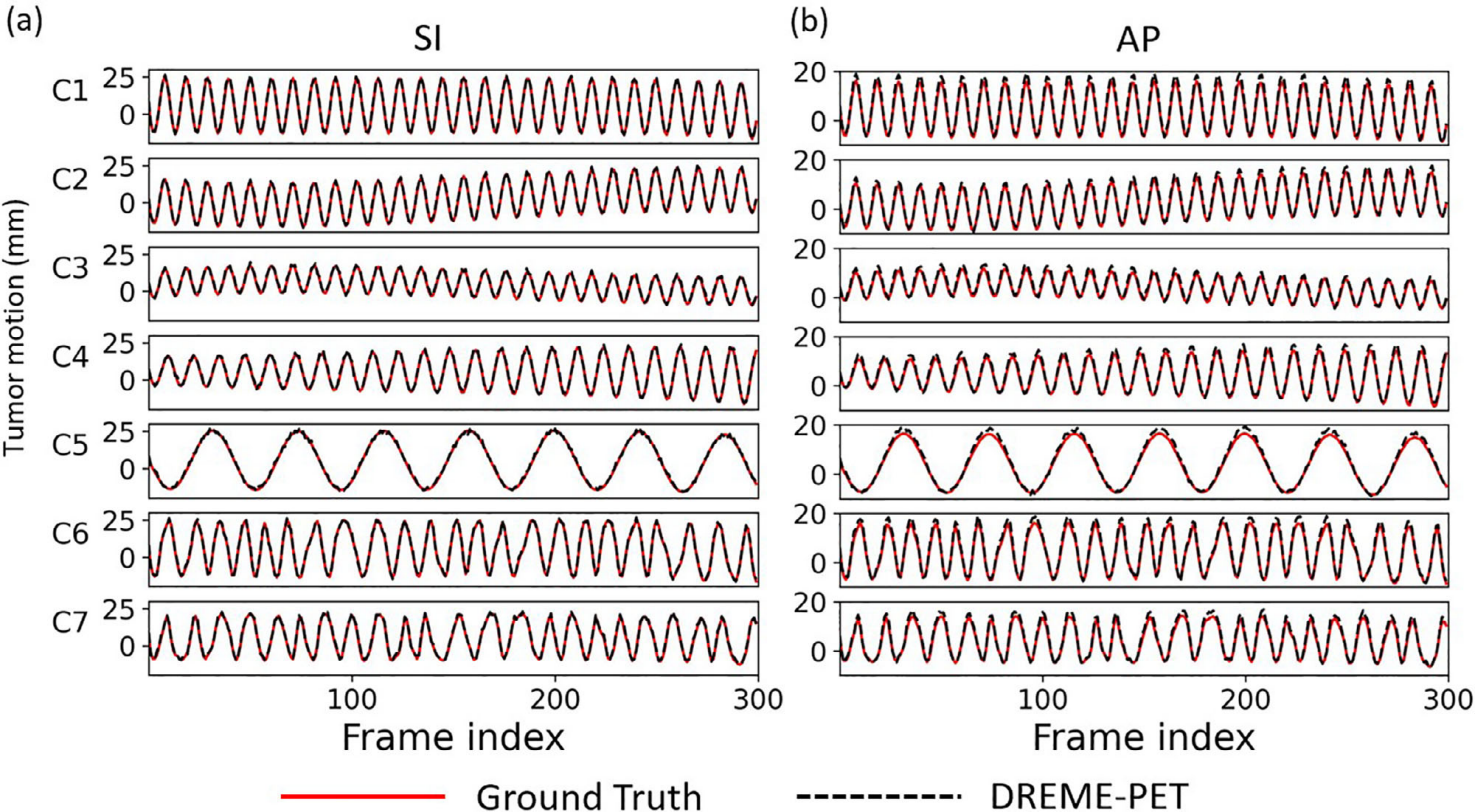
Tumor motion trajectories solved by DREME‐PET across different scenarios (C1‐C7) along (a) superior‐inferior (SI) and (b) anterior‐posterior (AP) directions. For the C2 scenario, the results show the solved motion via time‐resolved reconstruction, as the DREME‐PET model was trained using all the raw data of C2. For the other scenarios (C1, C3–C7), the results show the solved motion via real‐time predictions, as the motion was directly inferred based on the model trained on C2.

**FIGURE 4:**
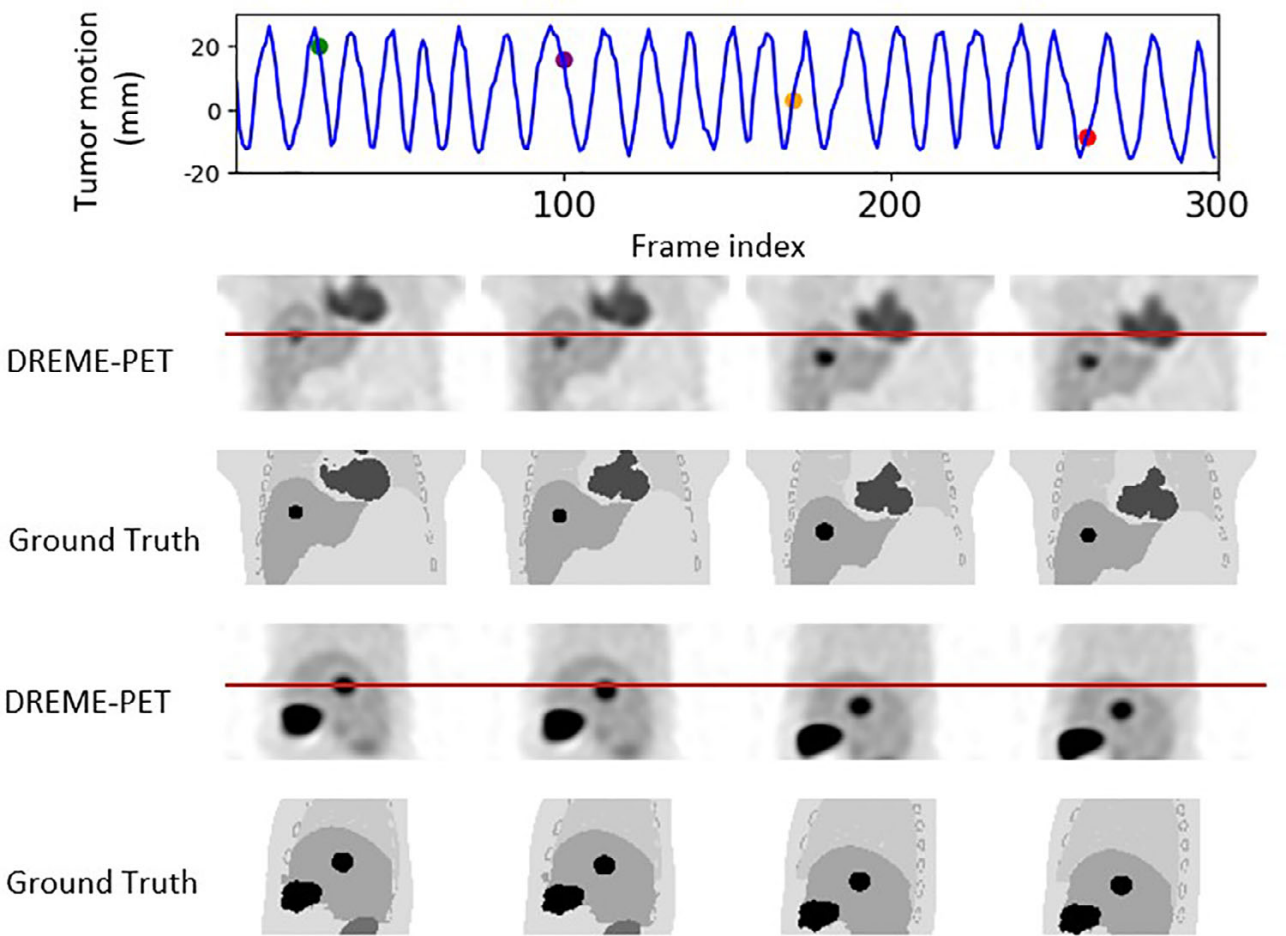
Example of real‐time PET images and tumor motion estimated by DREME‐PET for the C6 scenario of the XCAT study. The DREME‐PET model was trained on the C2 motion scenario and directly applied to C6 for real‐time imaging/motion prediction. The first row shows the solved tumor motion along the SI direction, with dots marking the four selected time points. The second and fourth rows present the DREME‐PET reconstructions in the coronal and sagittal views, while the third and fifth rows show the corresponding “ground‐truth” images.

To demonstrate the motion‐tracking accuracy of DREME‐PET, the solved tumor motion trajectories of DREME‐PET for all scenarios (C1‐C7) are presented in Figure 3. The motion tracked along superior‐inferior (SI) and anterior‐posterior (AP) directions are shown in Figure 3(a,b), respectively. DREME‐PET produces smooth trajectories that closely follow the “ground truth” with minimal errors. As noted above, the results from C2 scenario reflect the tracking accuracy of time‐resolved reconstruction, while the other six scenarios evaluate the tracking accuracy of real‐time predictions. To further highlight DREME‐PET's real‐time imaging/motion prediction performance, Figure 4 provides a representative example of real‐time PET images estimated by DREME‐PET for the C6 scenario of the XCAT study. Four timeframes are selected to illustrate different motion states in the coronal and sagittal views. It can be observed that real‐time DREME‐PET matches well with the “ground truth”.

To further demonstrate the robustness of DREME‐PET, particularly in the presence of tracer activity decay, we conducted two parameter studies using different numbers of events during training and testing. In both studies, the model was trained on scenario C2 and evaluated across all seven scenarios. The COME and DSC metrics were used for evaluation, and the results are summarized in Table 5. In the first paramter study, the model was trained and tested using the same number of events per time frame, with values ranging from 0.1 to 1.0 million. As expected, motion‐tracking performance improved with more training events, as more events per time frame provide a better estimate of the patient activity distribution $I_{ref}$, and all configurations achieved sub‐voxel‐level accuracy. In the second study, the model was trained with 0.2 million events per time frame but tested using reduced event counts ranging from 60% to 100%. With a robust CNN‐based motion encoder, motion‐tracking performance remained consistent across all cases. Note that the half‐life of FDG is approximately 110 min, while an averaged PET‐LINAC treatment session, including patient setup time, pre‐delivery imaging time, and beam‐on time, was 19.5 min for all treatment regions.^60^ Therefore, DREME‐PET is capable of handling the effects of tracer activity decay during the imaging process.

**TABLE 5:** Center‐of‐mass error (COME) and dice similarity coefficient (DSC) as accuracy metrics of two parameter studies.

Parameter study 1: DREME‐PET trained and tested using same numbers of events per time frame	**Number of events used for training and testing**	**COME (mm) ↓** **DSC ↑**
	0.1 million	3.0 ± 1.5 0.821 ± 0.066
	0.2 million	1.7 ± 0.8 0.885 ± 0.037
	0.5 million	1.6 ± 0.9 0.895 ± 0.034
	1.0 million	1.3 ± 0.6 0.901 ± 0.028
Parameter study 2: DREME‐PET trained with 0.2 million events per time frame, and tested with reduced numbers of events.	**Number of events used for testing**	**COME (mm) ↓** **DSC ↑**
	60% × 0.2 million	2.0 ± 1.0 0.859 ± 0.043
	70% × 0.2 million	2.0 ± 1.0 0.860 ± 0.049
	80% × 0.2 million	1.9 ± 0.9 0.862 ± 0.048
	90% × 0. 2 million	1.9 ± 0.9 0.865 ± 0.043
	100% × 0.2 million	1.7 ± 0.8 0.885 ± 0.037

*Note*: Both studies are trained on the C2 scenario and evaluated across all seven scenarios. The arrows point to the direction of improved accuracy.

### 3.2 The physical phantom study

**FIGURE 5:**
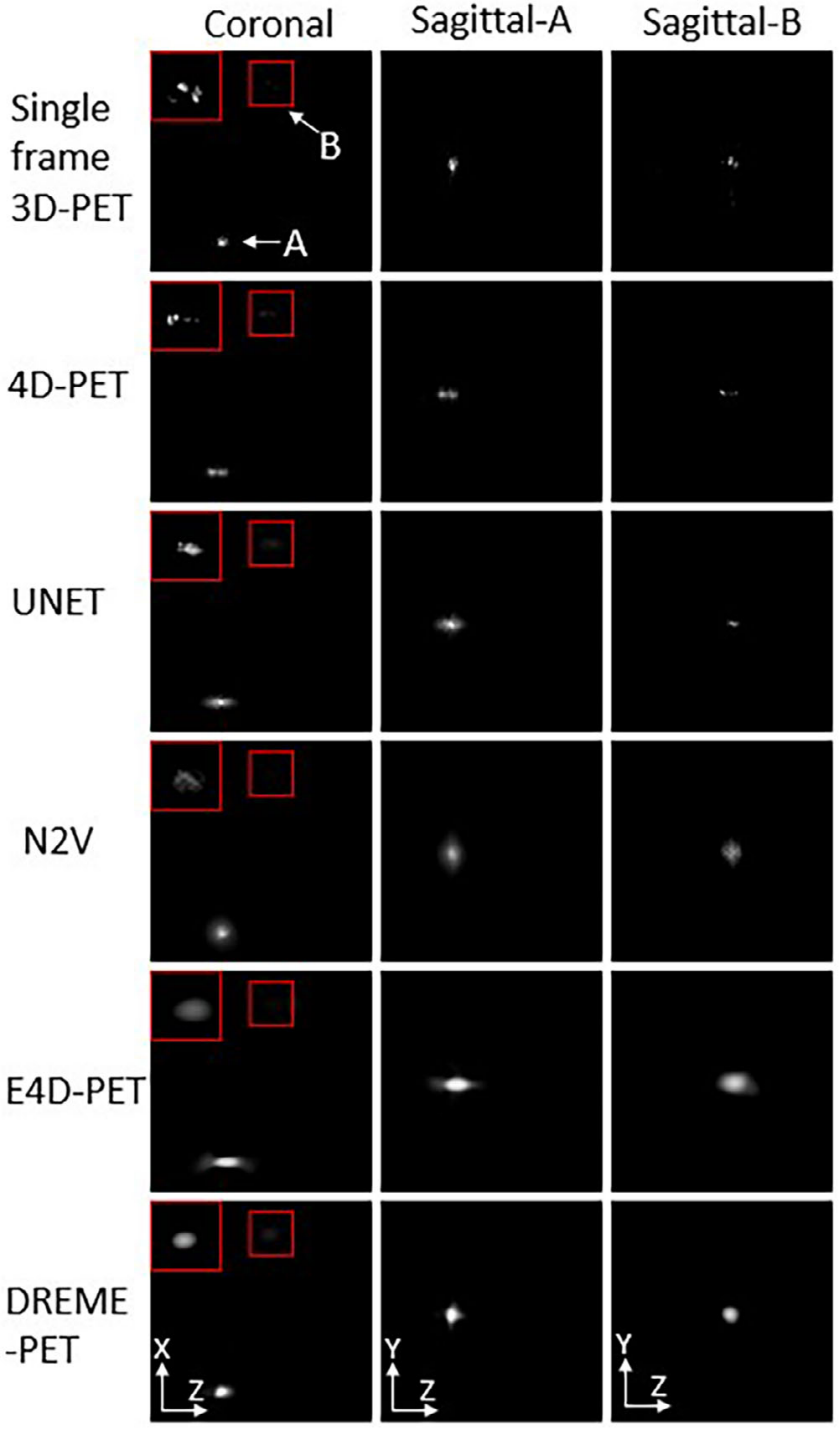
Comparison of reconstructed PET images at one frame from scenario S3 of the physical phantom study, between single‐frame 3D‐PET, 4D‐PET, UNET, Noise2Void(N2V), E4D‐PET and DREME‐PET. The first column shows the coronal view, with an inset highlighting the target B with an adjusted window level in each image. The second and third columns show the sagittal views of target A and B, respectively. The window levels for target A and target B were set to [0,10] and [0, 1.6], a.u., respectively. For 4D‐PET and E4D‐PET, the image with its phase matching that of the shown time frame is selected for display and comparison.

Figure 5 shows a representative frame of time‐resolved physical phantom PETs of scenario S3, reconstructed using different methods. The phantom contains two point sources moving along the longitudinal (z) direction. In the first column, the coronal view (x‐z plane) of the phantom is shown, with the higher‐intensity source labeled as target A and the other as target B. The sagittal views (y‐z plane) of targets A and B are presented in the second and third columns, respectively. To highlight the details of target B, an inset is added with an adjusted window level to better display its activity distribution. For 4D‐PET and E4D‐PET, the image with its phase matching that of the shown time frame is selected for display and comparison. In the single‐frame 3D‐PET, target B is difficult to observe as only around 15% of total events were associated with target B. In the 4D‐PET results, both targets suffer from residual motion‐related artifacts. Similarly, as UNET was trained using 4D‐PET results as the reference, its images showed similar artifacts. The results of N2V shows reduced noise compared with single‐frame 3D‐PET images, but target B's boundary remained difficult to discern, particularly in the x‐z view. The E4D‐PET reconstruction also suffers from motion‐related artifacts affecting both targets, especially for target A in the x‐z view, resulting in a blurry boundary. In contrast, the DREME‐PET results present both targets with reduced artifacts, by making use of all available counts while solving a time‐resolved motion model during the reconstruction.

Table 6 included the quantitative results of the physical phantom study, using three metrics across different reconstruction methods. The DREME‐PET model was trained on S3 and evaluated on all four motion scenarios (S1‐S4). Thus, S3 results indicate the accuracy of time‐resolved reconstruction, and the others indicate the accuracy of real‐time imaging/motion predictions. To assess the motion accuracy, the motion estimated by each method was compared with the programmed reference motion curves using COME and the Pearson correlation coefficient. Both targets were considered, and the motion extracted from them was averaged to obtain the result for each method. Due to the simple structure of the targets, single‐frame 3D‐PET, U‐Net, and Noise2Void can achieve 1–2 mm tracking accuracy, depending on the motion complexity. Since they rely on only one single frame as input, their image contrast is more affected by noise, resulting in a high relative error in contrast. The 4D‐PET, which uses sorted multi‐frame inputs, achieves better image contrast. However, its tracking accuracy is poor in scenarios S3 and S4, where irregular motion (Table 2) hinders the performance of sorting‐based methods. Similarly, E4D‐PET is also affected by irregular motion in S3 and S4, but it provides slightly improved image contrast compared with 4D‐PET. DREME‐PET outperforms other methods in all three metrics. All Wilcoxon signed‐rank tests of three metrics between DREME‐PET and other methods yielded *p* values < 10^−3^, confirming the statistical significance of DREME‐PET's better image reconstruction and motion tracking accuracy.

**TABLE 6:** Center‐of‐mass error (COME), Pearson correlation, and relative error of contrast as accuracy metrics for the physical phantom study.

Scenario	Single Frame 3D‐PET COME (mm)↓ Correlation ↑ $RE_{Contrast}$↓	4D‐PET COME (mm)↓ Correlation ↑ $RE_{Contrast}$↓	UNET COME (mm)↓ Correlation ↑ $RE_{Contrast}$↓	Noise2Void COME (mm)↓ Correlation ↑ $RE_{Contrast}$↓	E4D‐PET COME (mm)↓ Correlation ↑ $RE_{Contrast}$↓	DREME‐PET COME (mm)↓ Correlation ↑ $RE_{Contrast}$↓
S1	1.4 ± 1.2	1.5 ± 1.1	1.3 ± 1.5	1.0 ± 1.5	1.4 ± 1.4	**0.5 ± 0.4**
	0.985	0.969	0.967	0.978	0.965	**0.996**
	27.1% ± 24.0%	9.9% ± 5.2%	15.3% ± 13.7%	28.0% ± 36.9%	9.5% ± 5.9 %	**7.4% ± 6.3%**
S2	0.9 ± 1.0	1.4 ± 0.6	1.4 ± 1.0	0.9 ± 2.5	1.3 ± 1.0	**0.6 ± 0.4**
	0.985	0.980	0.974	0.938	0.968	**0.994**
	27.8% ± 24.0%	10.8% ± 6.8%	15.7% ± 12.3%	29.3% ± 33.3%	10.0% ± 4.2 %	**7.9% ± 5.7%**
S3	1.0 ± 2.3	3.3 ± 1.8	1.5 ± 1.1	0.8 ± 1.7	3.5 ± 2.1	**0.4 ± 0.4**
	0.960	0.884	0.974	0.977	0.891	**0.998**
	26.6% ± 23.2%	10.4% ± 4.4%	15.7% ± 11.8%	25.0% ± 34.7%	9.8% ± 3.8 %	**7.1% ± 6.4%**
S4	1.2 ± 1.2	4.6 ± 2.1	1.5 ± 1.8	0.8 ± 1.7	4.5 ± 2.2	**0.6 ± 0.6**
	0.993	0.781	0.953	0.977	0.787	**0.996**
	29.5% ± 23.3%	11.4% ± 14.2%	16.7% ± 17.4%	30.1% ± 38.5%	10.3% ± 6.0 %	**7.1% ± 6.3%**

*Note*: For the S3 scenario, the results quantify the motion accuracy of time‐resolved reconstruction, as the DREME‐PET model was trained using all the raw data of S3. For the other scenarios (S1, S2, S4), the results quantify the motion accuracy of real‐time predictions, as the motion was directly inferred based on the model trained on S3. The arrows point to the direction of improved accuracy.

**FIGURE 6:**
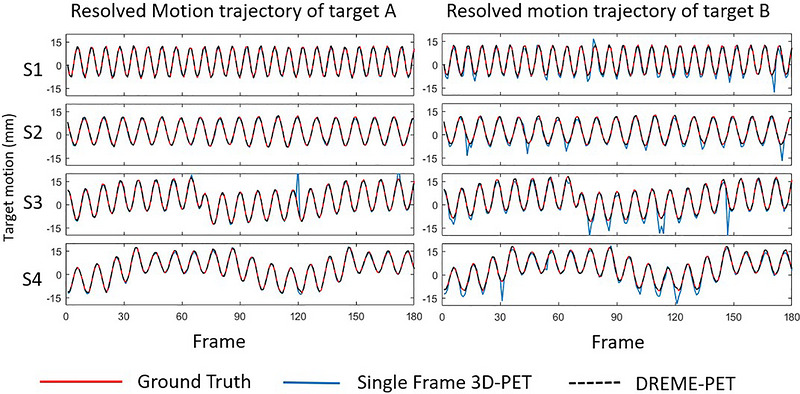
Comparison of target A and target B motion trajectories, which were generated from the single‐frame 3D‐PET, DREME‐PET, and “ground truth” for scenarios S1‐S4. As the DREME‐PET model was trained on scenario S3, S3 shows the time‐resolved reconstruction results, and the others show the real‐time motion prediction results.

**FIGURE 7:**
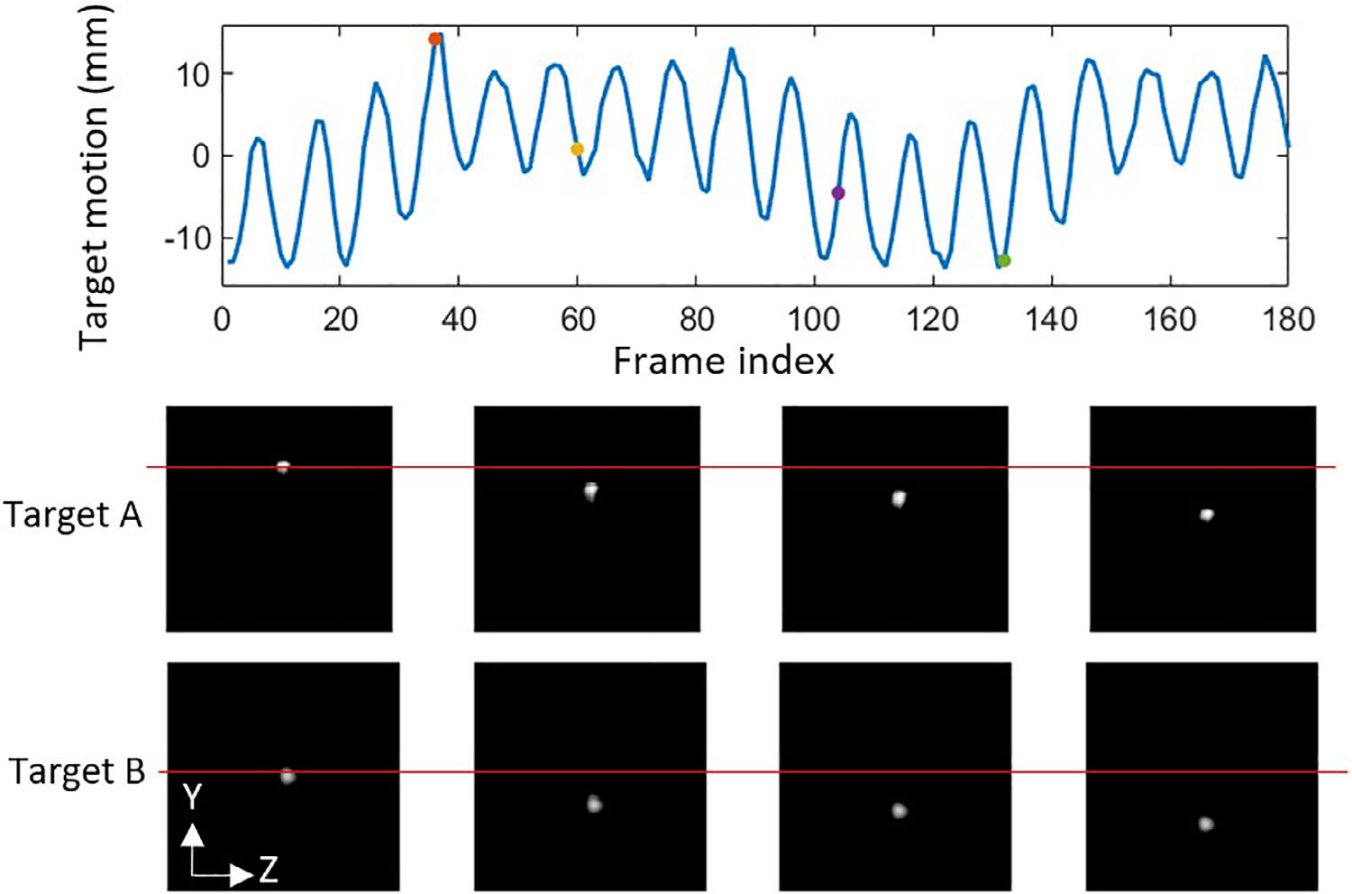
Example real‐time PET images and motion estimated by DREME‐PET for the S4 scenario of the physical phantom study. The first row shows the tracked target motion (averaged between targets A and B), with dots marking the four selected time points for plotting. Second and third rows are the corresponding sagittal views of targets A and B along the four time spots, respectively. The window levels for target A and target B were set to [0,10] and [0, 1.6], a.u., respectively.

Figure 6 compares the resolved motion trajectories of targets A and B using DREME‐PET and single‐frame 3D‐PET across all four scenarios, using the programmed motion curves as “ground truth”. As the DREME‐PET model was trained on scenario S3, S3 shows the time‐resolved reconstruction results, and the others show the real‐time motion prediction results. For target A, due to its relatively higher activity, both methods produce curves similar to the “ground truth”, with single‐frame 3D‐PET showing relatively more errors in S3. For target B, due to its limited number of events for MLEM reconstruction, 3D‐PET is more affected by noise, resulting in an inaccurate trajectory estimation. To better demonstrate the real‐time PET imaging/motion prediction accuracy, Figure 7 presents an example of the real‐time PET images in scenario S4. The first row shows the motion curve solved by DREME‐PET, from which four time spots were selected for plotting. The corresponding sagittal view of target A and target B are shown in the second and third rows of Figure 7. The motion of both sources matched well with the programmed reference trajectory.

In addition, we conducted cross‐dataset validation on the phantom study to evaluate the generalization ability of the proposed framework. For each experiment, the model was trained on one scenario and evaluated on all four scenarios, and the results are summarized in Table 7. Across all experiments, the method achieved sub‐voxel COME and a correlation coefficient greater than 0.99. The relative errors of the image contrast ranged from 4% to 8%. These results demonstrate DREME‐PET's capability as a real‐time PET imaging and motion estimation framework, and its robustness of under varying motion patterns.

**TABLE 7:** Cross‐validation results of the physical phantom study among four scenarios. The training scenarios are shown in columns while the testing ones are shown in rows.

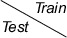	S1 (Training) COME (mm) ↓ Correlation ↑ $RE_{Contrast}$↓	S2 (Training) COME (mm) ↓ Correlation↑ $RE_{Contrast}$↓	S3 (Training) COME (mm) ↓ Correlation↑ $RE_{Contrast}$↓	S4 (Training) COME (mm) ↓ Correlation↑ $RE_{Contrast}$↓
**S1 (Testing)**	0.4 ± 0.3	0.6 ± 0.4	0.5 ± 0.4	0.7 ± 0.6
	0.997	0.996	0.996	0.993
	6.8% ± 4.0%	4.5% ± 3.1%	7.4% ± 6.3%	5.4% ± 4.4%
**S2 (Testing)**	0.6 ± 0.4	0.5 ± 0.4	0.6 ± 0.4	0.7 ± 0.6
	0.995	0.995	0.994	0.993
	6.8% ± 3.8%	4.6% ± 3.2%	7.9% ± 5.7%	4.9% ± 3.7%
**S3 (Testing)**	0.8 ± 0.8	0.8 ± 0.7	0.4 ± 0.4	0.4 ± 0.3
	0.991	0.994	0.998	0.998
	6.3% ± 4.6%	4.3% ± 3.2%	7.1% ± 6.4%	4.3% ± 4.0%
**S4 (Testing)**	0.9 ± 0.7	0.8 ± 0.7	0.6 ± 0.6	0.5 ± 0.4
	0.991	0.994	0.996	0.998
	6.2% ± 4.2%	4.7% ± 3.3%	7.1% ± 6.3%	3.8% ± 3.8%
**Mean**	0.7 ± 0.6	0.7 ± 0.6	0.5 ± 0.5	0.6 ± 0.5
	0.994	0.995	0.996	0.996
	6.5% ± 4.1%	4.5% ± 3.2%	7.4% ± 6.1%	4.6% ± 4.0%

*Note*: The arrows point to the direction of improved accuracy.

**FIGURE 8:**
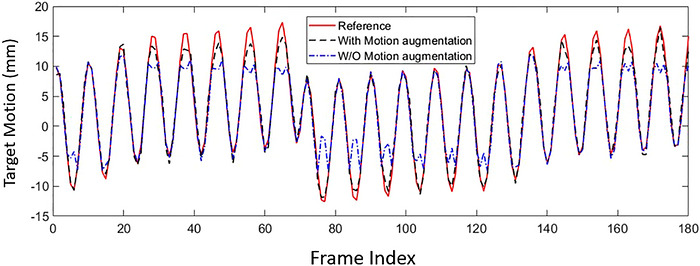
Comparison of motion tracking performance of the DREME‐PET model trained with and without motion augmentation.

Note that the DREME‐PET models trained on S1 and S2 also perform well on S3 and S4, even though the motion ranges of S1 and S2 are only 20 mm, while those of S3 and S4 are 30 mm (Table 2). This improvement is attributed to the motion‐augmentation procedure in training stage II‐d (Section 2.3.2). In this stage, random MBC scores are generated to create unseen motion states, enabling the CNN motion encoder to capture a broader range of motion variations. A comparison was conducted using the DREME‐PET model trained on scenario S1 and then evaluated on S3, with and without the motion‐augmentation stage. The motion curves extracted from both models are shown in Figure 8. Without augmentation, the estimated motion is constrained around the original training dataset's motion range of −10 mm to 10 mm. In contrast, with motion augmentation, the model is able to track motion beyond ±10 mm, demonstrating the effectiveness of the motion augmentation strategy.

### 3.3 The patient study

**FIGURE 9:**
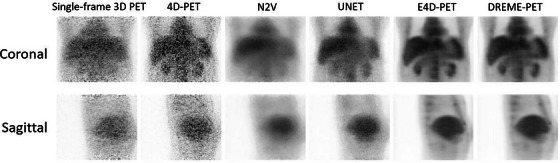
Comparison of time‐resolved PET reconstruction for a single frame in the patient study, across single‐frame 3D‐PET, 4D‐PET, UNET, Noise2Void(N2V), E4D‐PET, and DREME‐PET. The first and second rows show the coronal and sagittal views, respectively. For 4D‐PET and E4D‐PET, the image with its phase matching that of the shown time frame is selected for display and comparison.

Figure 9 shows a representative frame of time‐resolved patient PET reconstructions, across different methods in the coronal and sagittal views. For 4D‐PET and E4D‐PET, the image with its phase matching that of the shown time frame is selected for display and comparison. In the single‐frame 3D‐PET, the boundaries of the liver and other organs are difficult to discern due to the insufficient number of events (0.3 million per time frame). With phase sorting and binning, the 4D‐PET provides higher image contrast, but the structural clarity remains limited. The images of UNET and N2V exhibit reduced noisy compared to non‐learning methods, but the structure boundaries are still unclear. E4D‐PET achieves improved image quality by fully utilizing the measured events through phase‐based motion modeling. In contrast, DREME‐PET displays the sharpest structural details and the least motion artifacts. Note that E4D‐PET achieves results comparable to DREME‐PET because the motion in this patient is relatively regular; its performance is expected to be more challenging under irregular motion conditions, which will lead to phase sorting errors and larger intra‐phase motion.

**FIGURE 10:**
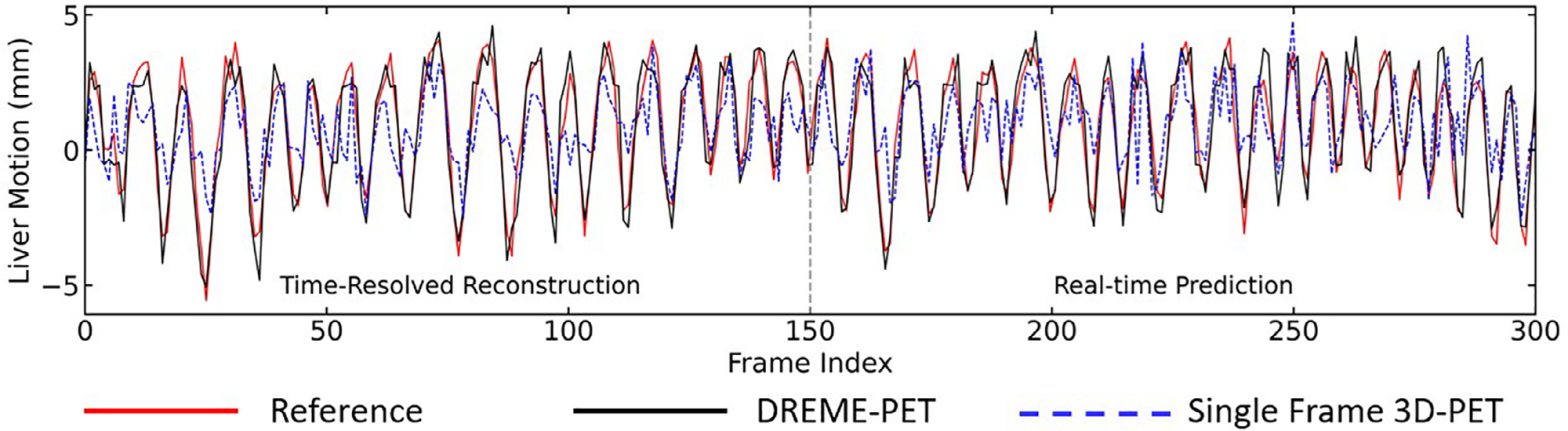
Liver motion trajectories solved by DREME‐PET and single‐frame 3D‐PETs. For DREME‐PET, the first 150 frames show the solved motion via time‐resolved reconstruction. For the following 150 frames, the results show the solved motion via real‐time predictions, as the motion was directly inferred based on the models trained on the first 150 frames. The reference curve shows the signals derived from the number of events, used as a surrogate of respiratory motion.

**FIGURE 11:**
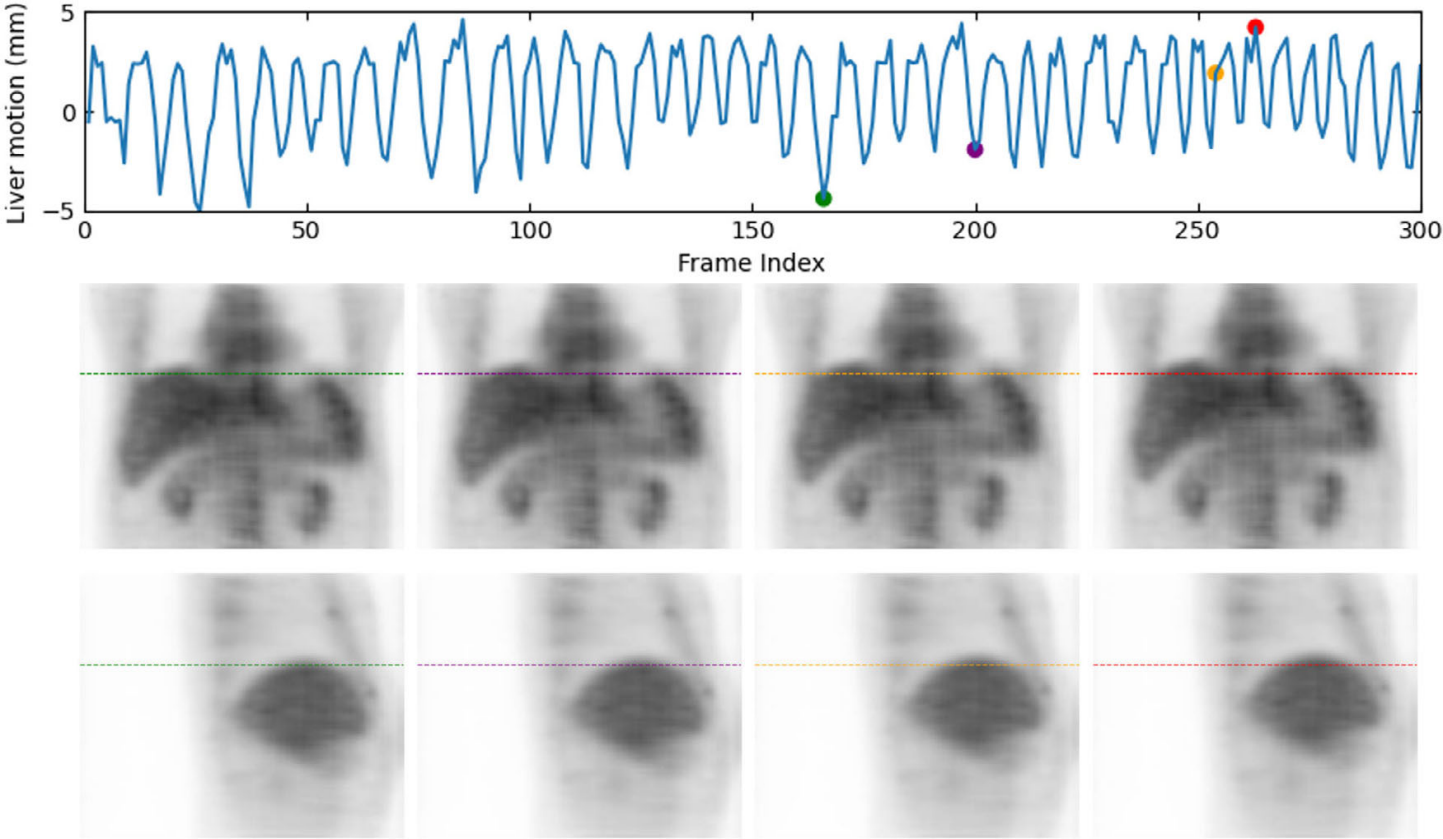
Example real‐time PET images and motion estimated by DREME‐PET of the patient study. The first row shows the resolved liver motion with dots marking the four selected time points for plotting. Second and third rows are the corresponding coronal and sagittal views of patient along the four time spots, respectively.

Figure 10 shows the resolved motion trajectories of the liver using DREME‐PET and single‐frame 3D‐PET of the patient study, with the motion derived from the decay‐corrected number‐of‐events curve serving as the reference for temporal trends. The reference signal is scaled to match the amplitude of the DREME‐derived motion and reflects more of a qualitative “ground truth”. Since the DREME‐PET was trained on the first 150 frames, the results from the remaining 150 frames represent the real‐time prediction performance. Due to the high noise level of single‐frame 3D PET, it shows larger fluctuations compared to DREME‐PET, leading to inferior motion tracking results. Notably, due to the natural decay of the tracer activity, the number of events per timeframe decreases over time. DREME‐PET benefits from its augmentation mechanism during training, which helps to mitigate the motion and count variations in the list‐mode data. To better illustrate the real‐time PET motion prediction result, Figure 11 shows an example of the real‐time time‐resolved PET results. The first row shows the motion curve estimated by the DREME‐PET, with four time spots selected to represent the different motion phases. The corresponding coronal and sagittal views of the patient along these time spots are shown in the second and third rows of Figure 11.

Table 8 presents the quantitative results of the patient study, evaluated using two metrics across all six reconstruction methods. The DREME‐PET was trained on the first 150 frames and then evaluated on all 300 frames, so the results from the first and second 150 frames represent the time‐resolved reconstruction and the real‐time prediction, respectively. For 4D‐PET and E4D‐PET, the first 150 frames were first sorted and binned into 10 phases, followed by reconstruction into 10 phase‐resolved PET images. The remaining 150 frames were then assigned to these phases, and the associated phase‐resolved PET images were used for comparison. The learning‐based methods, UNET and N2V, improved the performance over single‐frame 3D‐PET reconstruction by suppressing the noise. E4D‐PET significantly improves both metrics, compared with conventional EM‐based methods. This gain is primarily attributed to the use of all available events, which enhances image quality and, consequently, enables more accurate phase‐based motion estimation. In contrast, DREME‐PET achieves the best performance due to its finer temporal resolution, effectively assigning a distinct motion state to each frame, whereas E4D‐PET is limited to a fixed number of phases. This higher temporal resolution leads to clearer liver delineation, thereby enabling more accurate segmentation and motion tracking. All Wilcoxon signed‐rank tests across two metrics comparing the DREME‐PET with other methods yielded *p* values < 10^−3^, confirming the statistically significant improvement of DREME‐PET in image reconstruction and motion tracking accuracy.

**TABLE 8:** The Pearson correlation and relative error of contrast as accuracy metrics for the patient study.

	Single Frame 3D‐PET Correlation ↑ $RE_{Contrast}$↓	4D‐PET Correlation ↑ $RE_{Contrast}$↓	UNET Correlation ↑ $RE_{Contrast}$↓	Noise2Void Correlation ↑ $RE_{Contrast}$↓	E4D‐PET Correlation ↑ $RE_{Contrast}$↓	DREME‐PET Correlation ↑ $RE_{Contrast}$↓
First 150 frames	0.62 19.4% ± 4.9%	0.65 14.6% ± 6.5%	0.68 7.3% ± 4.3%	0.67 6.1% ± 3.1%	0.83 4.8% ± 2.3%	**0.94** **3.6% ± 0.4%**
Second 150 frames	0.61 20.2% ± 4.5%	0.70 14.3% ± 6.5%	0.72 6.1% ± 4.2%	0.70 6.1% ± 4.2%	0.80 5.0% ± 2.3%	**0.91** **3.6% ± 0.4%**

*Note*: The first and second 150 frames indicate the time‐resolved reconstruction and real‐time prediction of DREME‐PET, respectively.

## 4 DISCUSSION

In this study, we proposed a dual‐task framework, DREME‐PET, for time‐resolved PET reconstruction and real‐time PET/motion prediction. Clinically, time‐resolved PET provides accurate tumor localization and motion characterization, enabling patient‐specific motion management strategy development and optimization. The real‐time imaging and motion estimation capability, on the other hand, enables real‐time motion monitoring, treatment adaptation, and dose verification for PET‐guided radiotherapy. DREME‐PET adopts a “one‐shot” learning strategy, eliminating the need for large external datasets for model training. It can utilize all list‐mode data from a conventional PET scan for both patient activity map reconstruction and motion modeling, thereby avoiding potential biases introduced by population‐based models. For radiotherapy, it enables the derivation of a patient‐specific model from a setup PET scan of each treatment fraction (via PET‐LINAC). Subsequently, the model can be used for same‐fraction real‐time imaging, motion monitoring, and treatment adaptation, minimizing uncertainties from outdated activity maps/motion models. By integrating time‐resolved PET reconstruction and real‐time imaging into a unified framework, DREME‐PET enables more efficient model training and deployment within clinical workflows. During real‐time imaging, it directly uses list‐mode data as input. With the count‐ and motion‐augmentation strategy (Section 2.3.2), it is robust to variations in count rates and motion amplitudes (Tables 5 and 7), allowing reliable performance even during long scans when tracer activity decay is significant. DREME‐PET was validated using an XCAT simulation study and further tested on a physical phantom study and a patient study. The XCAT study demonstrated the effectiveness of the “one‐shot” training strategy, capturing motion variations with irregular respiratory patterns in both time‐resolved reconstruction and real‐time prediction. In the physical phantom study, DREME‐PET achieved a low COME of approximately 0.7 mm, a high Pearson correlation coefficient (>0.99), and a relative error in contrast below 8%. In the patient study, DREME‐PET maintained a high Pearson correlation coefficient (≥0.91) and achieved a relative error in contrast below 4%, demonstrating its robustness under realistic imaging conditions. The results indicate that DREME‐PET is robust to unseen real‐time motion scenarios, owing to the motion‐augmentation‐enhanced generalizability of the CNN motion encoder. Among the comparison methods, E4D‐PET achieves image quality comparable to that of DREME‐PET. However, as a sorting‐driven approach based on predefined phase binning, it lacks robustness in the presence of irregular motion, and it is not designed to accurately track real‐time, varying motion. In contrast, DREME‐PET estimates motion in a continuous, frame‐wise manner, allowing for real‐time operation and improved robustness under irregular motion conditions.

DREME‐PET could achieve high reconstruction accuracy with desired computational efficiency. A typical processing time for the list‐mode data of one timeframe is 28 ms. Specifically, one list‐mode MLEM iteration requires 14.6 ms, while the CNN‐based motion encoder inference takes 5.2 ms. The deformation vector field computation and its application to the image volume require 0.5 ms. The remaining 7.7 ms are attributed to data input/output and preprocessing operations. For BgRT, which typically operates at a temporal resolution of 100 ms^43^ (Reflexion uses a sliding window design that incorporates mixed prior/new signals of 500 ms for each frame), the proposed framework is sufficiently fast to achieve real‐time motion estimation. To convert the list‐mode raw data to PET volumes for motion feature extraction, we used one‐iteration MLEM instead of conventional filtered back‐projection (with additional post‐filtering), due to MLEM's efficiency and data modeling advantage. In our experiment, converting list‐mode data containing 0.2 million events into a sinogram takes approximately 24 ms, and the back‐projection and post‐filtering take around 66 ms. The efficiency discrepancy is because sinogram‐based back‐projection processes all possible lines of response (LORs) in the projection space, whereas list‐mode‐based methods operate only on the recorded LORs in the list‐mode data. Moreover, owing to its more accurate physical modeling, MLEM can incorporate time‐of‐flight modeling when desired, providing greater flexibility and applicability across different imaging scenarios and scanner configurations.

The runtimes of comparison methods are also recorded as a comparison. In our XCAT simulation experiment, the runtime of the EM‐based method depends on the size of the list‐mode data and the number of iterations. In our setup, a typical single‐frame 3D PET reconstruction needs 14.6 ms. The end‐to‐end runtime of learning‐based methods (UNET and Noise2Void), including the reconstruction time of the original noisy images (single‐frame 3D‐PET) and DL post‐processing, is approximately 23.6 ms. Although these methods could yield fast inference, the structural fidelity and tracking accuracy are limited in the challenging low‐count scenarios. For 4D‐PET, after phase sorting, the reconstruction time is 265.1 ms per phase due to more list‐mode data being involved. For E4D‐PET, the intensive motion modeling and forward‐backward iterations require a substantially longer time (∼45 min). Both 4D‐PET and E4D‐PET, however, are retrospective‐sorting‐based techniques and not readily applicable for real‐time imaging. Thus, the real‐time inference runtime is not reported. Nonetheless, if the real‐time list‐mode data can be assigned/matched to a phase of the reconstructed 4D images, it can be extended to real‐time imaging (without considering potential motion variations, though), and the latency is mostly determined by the phase assignment/matching process (likely a few ms).

In our physical phantom study, we used two‐point sources as targets. Due to memory constraints, we used only a subset of the list‐mode data in each time frame by limiting the maximum ring difference, allowing the data to be converted into a smaller sinogram. Single‐slice re‐binning^61^ or Fourier re‐binning^62^ could be used to build a small and dense sinogram in clinical data. Another potential approach is to use only a subset of the sinogram in each iteration, similar to what is done in OSEM.

In the patient study, a subset of the list‐mode data was used in each timeframe by limiting the maximum ring difference. In addition, inspired by OSEM, only half of the sinogram was used in each iteration for loss calculation to accelerate training and reduce GPU memory use. Despite these data reductions, the proposed method demonstrated strong and consistent performance in the patient study, in the presence of more complex anatomical structures and motion patterns. These results demonstrate the robustness of the proposed framework under realistic imaging conditions when using such subset data. Incorporating the full list‐mode data is expected to further improve the reconstruction accuracy, should more advanced hardware become available. For attenuation correction, a static attenuation correction CT (AC‐CT) was incorporated into the reconstruction process. In our XCAT study, we used an attenuation map simulated based on first timeframe and applied it uniformly to attenuate‐correct all timeframes. For the phantom study, attenuation correction was not applied because photon attenuation is neglibgible in this simple two‐point‐source setup. For our patient study, the static AC‐CT was obtained from the PET/CT system, rigidly registered to the PET reference image, and then incorporated into all timeframes reconstruction. Accurate registration between the attenuation map and the PET image is critical in clinical practice, as it directly affects the accuracy of SUV quantification.^63^ More advanced approaches, such as mutual information‐guided iterative methods,^64^ and deep learning–based deformable registration methods,^63^, ^65^, ^66^ could further improve alignment. Deep learning methods can better handle differences in intensity distributions between modalities and generally outperform conventional approaches, which are often computationally expensive and sensitive to image noise.^66^ In addition, the activity/attenuation map registration in our study does not consider the variation induced by patient motion.^63^ To address this, one possible solution is to first register the attenuation map with the reference activity map, and then propagate the aligned attenuation map to other motion states using the intra‐scan DVFs concurrently estimated by DREME‐PET for attenuation correction.

In this work, the XCAT study, the physical phantom experiment and the patient study were performed using diagnostic PET systems. Future works can evaluate the DREME‐PET framework on the RefleXion PET‐LINAC as well. The implementation of BgRT could benefit from DREME‐PET from the following aspects: (1) Due to the concerns of the PET signal decay during the treatment, the BgRT timer mandates that the BgRT delivery occurs within 1 h of the PET pre‐scan to ensure a safe delivery.^67^ The count‐augmented CNN motion encoder of DREME‐PET can account for such activity decays, allowing the treatment course to be extended if necessary. (2) Due to the limited longitudinal field of view and detector sensitivity of the current Reflexion platform, the PET uptake in patients usually presents unclear structural boundaries with elevated heterogeneity,^67^ challenging the precision of PET‐guided delivery. In our DREME‐PET framework, the MLEM reconstruction of the raw data is not directly used for tracking, but fed into a CNN‐based motion encoder to determine the motion state of each time frame. Coupled with the latest motion model derived from a pre‐delivery PET scan, our strategy can yield more stable and reliable tumor localization results. Nevertheless, the implementation of DREME‐PET on the RefleXion system still requires further validation. Future studies that use more complicated, deformable motion‐enabled phantoms, along with comprehensive patient studies, can help to thoroughly assess the accuracy and limitations of DREME‐PET.

In other DREME‐series methods for CBCT^41^ and MRI^42^ reconstructions, a loss term called the zero‐mean loss is used. This loss forces the mean of the MBC scores across all time frames to be zero, removing non‐zero baselines. Removing such baselines helps to facilitate the motion augmentation (Equation 7), as the same MBC scaling factor can be used to equally augment the motion in both directions. In DREME‐PET, the MBC score is derived by the CNN motion encoder, using list‐mode data‐reconstructed MLEM images as input. Calculating the MBC scores for all time frames to derive the zero‐mean loss in each iteration would be time‐consuming. In our case, the running time for training stage I‐b is 3.6 s without the zero‐mean loss and 6.6 s with it. Thus, in this study, we eliminated this loss term during training to speed up the process, which did not affect the overall accuracy of time‐resolved reconstruction and real‐time image/time prediction. For the learnable B‐spline interpolant used to generate MBC, we adopt a hierarchical design to capture both smooth global motion and localized, potentially discontinuous motion. For more extreme non‐smooth motion patterns, performance could be further improved by using finer control point spacing or incorporating physics‐informed motion regularization, such as Jacobian loss^68^ to better represent complex motion dynamics. Our recent studies also demonstrated that more advanced representation strategies, such as the Gaussian representation^69^ could also help with better capturing complex motion scenarios like the sliding motion. Other regularization terms, such as the DVF self‐consistency loss^54^ could be potentially added to further enhance DREME‐PET, although the balance between the accuracy enhancement and the added computational complexity should be carefully considered. The training stage of the DREME‐PET is computationally more demanding than the subsequent inference stage. However, this training process is performed only once using the initial set of data. In a short timeframe (e.g., within a treatment fraction), the learned spatial motion/activity representation is expected to remain stable and consistent to enable real‐time inference. Between fractions, however, the activity/motion patterns may see larger discrepancies that warrant fraction‐specific learning. Learning each fraction from scratch will be time‐consuming and not clinically feasible based on current standard hardware. Recently, a prior‐adapted progressive training strategy was proposed for CBCT reconstruction based on the DREME framework.^70^ In this approach, a base DREME model is pre‐trained using pre‐treatment 4D‐CTs and then fine‐tuned with on‐treatment CBCT projections in a daisy chain scheme, reducing the training time to ∼10 min (compared to >1 h). Following the same principle, a simulation PET scan could be used to initialize the model, which can then be fine‐tuned using the pre‐treatment list‐mode data at each treatment fraction to expedite the per‐fraction training. In this way, DREME‐PET could adapt to inter‐fraction activity and/or motion variations efficiently, rendering it more clinically applicable. Additional acceleration strategies, including more efficient image/motion representation techniques,^69^ could also be explored. In addition, besides serving as guidance for activity map reconstruction^23^, ^28^ or attenuation map generation.^71^, ^72^ Chen et al. has shown that prior images like MRI can also assist with the respiratory motion correction of PET^73^ through derived motion fields. In the context of DREME‐PET, CT or MRI scans could be similarly used to help generate a patient‐specific motion model that can then be adapted by DREME‐PET, which warrants future investigations and validation.

## 5 CONCLUSION

We proposed DREME‐PET to jointly reconstruct a reference patient activity map and resolve a patient‐specific, data‐driven respiratory motion model. The derived motion model, learned through a motion encoder, enables real‐time volumetric PET imaging and respiratory motion tracking directly from low‐count PET list‐mode data. DREME‐PET is evaluated on an XCAT simulation study, a physical phantom study, and a patient study. The results demonstrate that the framework produces high‐quality images and highly accurate motion models with sub‐voxel precision to potentially benefit applications such as PET‐guided adaptive radiotherapy.

## CONFLICT OF INTEREST STATEMENT

The study was partially supported by funding from Varian Medical Systems.

## Supporting information



Supporting Information

## Data Availability

The data that support the findings of this study are available upon reasonable request from the authors.
