# Supplementary material for: Time‐resolved PET imaging using a joint dynamic reconstruction and motion estimation framework (DREME‐PET)
Source: Med Phys. 2026 Aug 4;53(8):e70593. doi: 10.1002/mp.70593 (PMC13438130; doi:10.1002/mp.70593)
Supplement: Supplementary file 1 [file MP-53-0-s001.pdf]

Supplementary Materials

Deformation Vector Field (DVF) example derived from DREME-PET

To illustrate the details of spatial sampling of the motion basis components in representing motion, we present displacement vector field (DVF) computed between the end-of-inhale and end-of-exhale positions from the patient study.

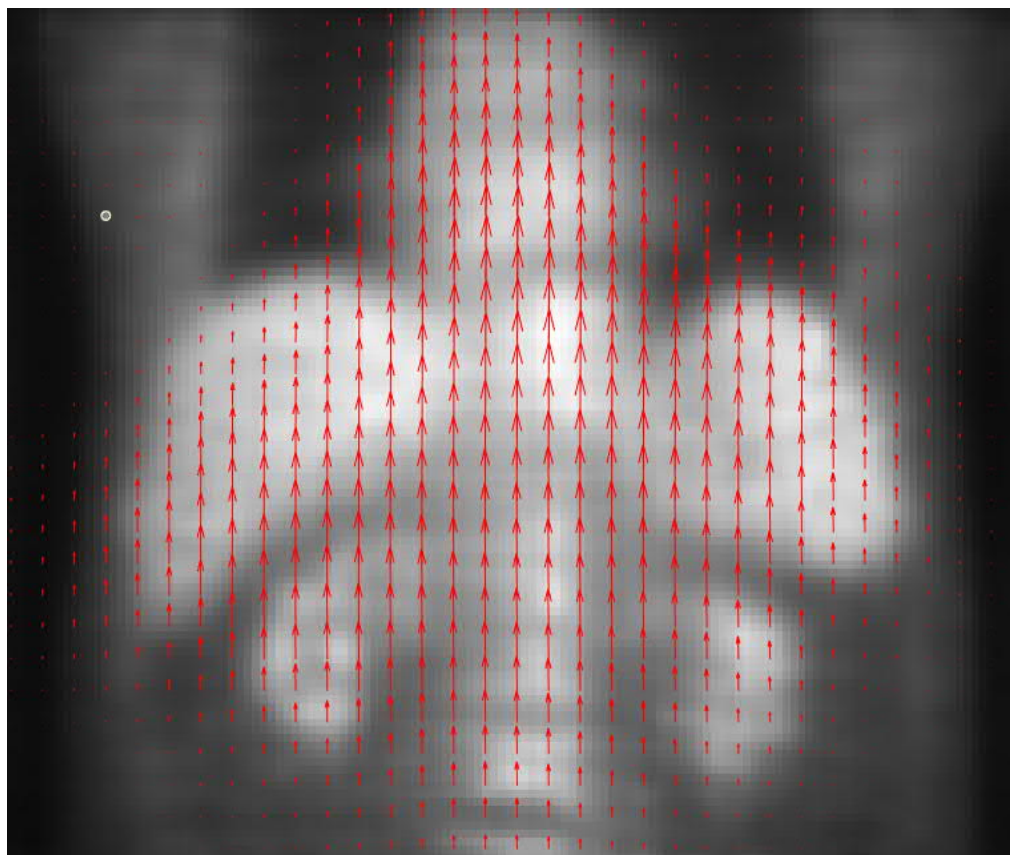

Figure S-1 Displacement vector fields generated by DREME-PET.

The results show that DREME-PET produces a smooth and spatially varying motion field, indicating that the learned basis components provide sufficiently fine spatial sampling to offer an accurate and physically consistent representation of the underlying motion.
